# Efficient Synthesis of Fused Polycyclic Ether Systems via Sulfonium Ylides: A Synthetic Approach to Yessotoxin and Adriatoxin

**DOI:** 10.3390/md23020051

**Published:** 2025-01-21

**Authors:** Federico Moya-Utrera, Iván Cheng-Sánchez, Irama Fuentes-Pino, Antonio Sánchez-Ruiz, Francisco Sarabia

**Affiliations:** 1 Department of Organic Chemistry, Faculty of Sciences, University of Málaga, 29071 Málaga, Spain; 2 Faculty of Pharmacy, University of Castilla-La Mancha, Campus de Albacete, Avda. Dr. José María Sánchez Ibáñez S/N, 02008 Albacete, Spain; antonio.sanchezruiz@uclm.es

**Keywords:** yessotoxin, adriatoxin, asymmetric epoxidation, stereoselective synthesis

## Abstract

A novel class of chiral sulfonium salts, derived from L- and D-methionine, was designed and successfully employed for the diastereoselective synthesis of epoxy amides. This new methodology of asymmetric epoxidation was exploited for the stereoselective construction of fused polycyclic ethers, which are structural motifs present in a great variety of natural products of marine origin. This methodology proved to be useful for the synthesis of the tricyclic **A–C** system contained in yessotoxin and adriatoxin, and also in many other related natural products of marine origin belonging to the fused polycyclic ether toxins.

## 1. Introduction

The unicellular algae dinoflagellates produce a particularly striking family of natural products characterized by ladderlike polycyclic ethers. The structural diversity of this class of natural products spans from relatively small molecules to the largest non-biopolymer substance known to date, such as maitotoxin [1,2]. These compounds are responsible for the red tide catastrophes that have led to mass deaths of fish and the poisoning of human beings along coasts all over the world. The structural complexity and potent biotoxicity of marine polyether toxins have propelled intense research activity in the synthetic and biochemical areas [3]. In general terms, molecular architectures are dominated by fusioned polytetrahydropyran systems interrupted by the presence of some more flexible seven-, eight-, or nine-membered rings. In consequence, many synthetic efforts have been devoted to designing suitable methodologies to construct these rigid frameworks. From a chemical standpoint, the challenges associated with their total syntheses have stimulated the development of efficient synthetic methodologies to construct the repetitive structural motifs present in these compounds. The daunting efforts carried out by different research groups in this direction have allowed the total synthesis of some of these compounds [4,5,6]. Particularly striking are the seminal contributions of Nicolaou’s group with the total synthesis of brevetoxins B and A in 1995 [7] and 1998 [8], respectively, as well as his notable contributions directed towards the total synthesis of maitotoxin [9]. Similarly important are the contributions of other research groups to the total synthesis of brevetoxins [10,11,12] and related marine toxins [13,14,15]. In general, these marine natural products exhibit similar biological activities characterized by neurotoxicity as a consequence of opening membrane protein ion channels that trigger an ion influx into cells [16].

**Figure 1:**
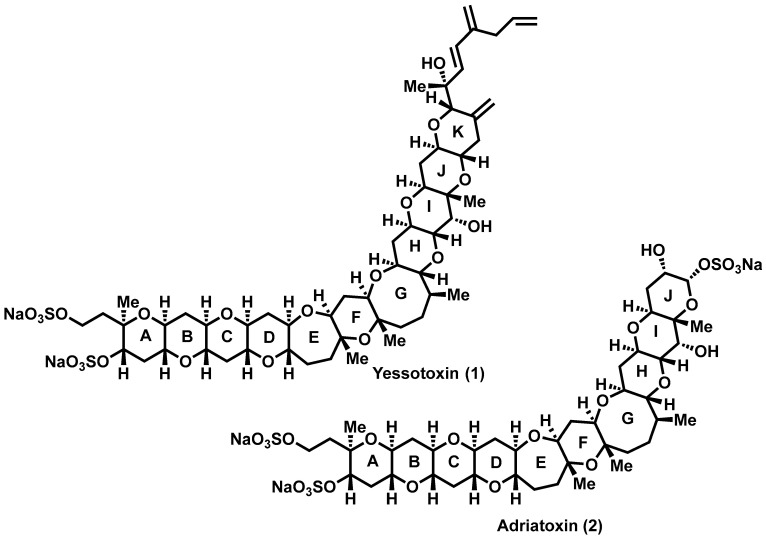
Molecular structures of yessotoxin (**1**) and adriatoxin (**2**).

Among the different ladderlike polyether biotoxins, yessotoxin (YTX, **1**) (Figure 1), initially isolated from dinoflagellate *Protoceratium reticulatum* in 1987 [17,18] and later from *Lingulodinium polyedrum* [19] and *Gonyaulax spinifera* [20], displays an intriguing biological activity that remarkably differs from other members of this large class of natural products. In fact, yessotoxin is considered a different “toxin” [21]. Thus, while initially included in the list of diarrhoetic shellfish poisoning (DSP), it had to be excluded later in this list as it was demonstrated that this compound did not cause such toxicity [22]. The reason for this relevant biological difference lies in the fact that its cellular target is not the sodium channel, despite its structural similarity to relevant phycotoxins such as brevetoxins, ciguatoxins, or gambierone, whose toxic effects are due to their interactions with this key protein. Particularly, it was disclosed that yessotoxin induces apoptosis through a mitochondrial signal transduction pathway, being the first time that a marine polycyclic ether was recognized as an antitumoral agent [23,24,25]. In addition, glycosylated yessotoxins, named protoceratins, were identified as highly potent cytotoxic agents against various cancer cell lines with IC values in the low nM range [26]. Further biological studies showed that YTX affects the cardiac muscle cells (creating intracytoplasmatic oedemas), modulates the calcium homeostasis in human lymphocytes, and induces selective disruption of the E-cadherin-catenin system in epithelial cells [27,28,29]. All these biological studies revealed that YTXs bind to phosphodiesterases (PDEs), leading to their activation. As a consequence, some metabolic pathways are activated through kinases PKC (Protein kinase C) and AKAPs (A-kinase anchor proteins) and can be explained its effects in calcium levels, cyclic adenosine monophosphate (cAMP), and mitochondrial conditions. Thus, the therapeutic applications of YTX for the treatment of cancer [30,31], Alzheimer’s disease, [32,33], allergy [34], or metabolic diseases [35] are being explored. In contrast, cardiotoxicity has been reported in rats as a consequence of exposure to YTX [29].

On the other hand, yessotoxin (**1**) is the parent compound of a family of various structurally related compounds named yessotoxins (YTXs), which are characterized by a series of 11 cyclic *trans*-fused rings, an unsaturated terminal chain, and two sulfated alcohol groups [36,37,38,39]. Interestingly, whereas many YTX analogs are biosynthetic products of algae, some derivatives are metabolic products of shellfish [40,41]. More recently, adriatoxin (**2**) was isolated from the digestive glands of DSP-infested mussels *Mytilus galloprovincialis* in the Adriatic Sea [42]. Closely related to yessotoxin, adriatoxin represents a truncated yessotoxin analog and contains the same A-J system of yessotoxin but lacks the K-ring and its corresponding side chain (Figure 1). Despite the intriguing biological profiles of YTX and related compounds, total synthesis of yessotoxin has not been described yet, although several synthetic approaches have been reported to address different fragments of its structure [43,44,45,46,47,48,49,50,51,52,53,54,55,56,57].

**Scheme 1:**
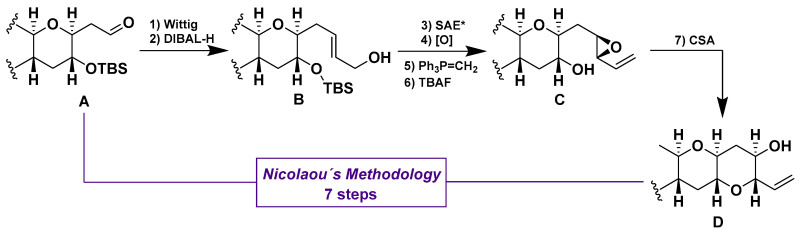
Synthetic strategy for the construction of polytetrahydropyran systems via the Nicolaou methodology (* SAE = Sharpless asymmetric epoxidation).

The first regio- and stereoselective synthesis of tetrahydropyrans contained in this class of molecules was reported by Nicolaou in 1985 based on an intramolecular ring opening reaction of a hydroxy vinyl epoxide [58]. In particular, starting from aldehyde type **A**, the synthesis of the key precursor, epoxy olefin **C**, would proceed in six steps via a tandem Wittig/Sharpless asymmetric epoxidation (SAE) through allylic alcohol **B**. Then, cyclation of epoxy olefin **C** was achieved under acidic conditions to afford tetrahydropyran D (Scheme 1). Related synthetic methodologies were efficiently developed by Mori, based on the use of sulfonyl-stabilized oxiranyllithium species [59], or by Jamison, based on a biomimetic cascade polyepoxide opening in aqueous media [60], among other authors [61].

**Scheme 2:**
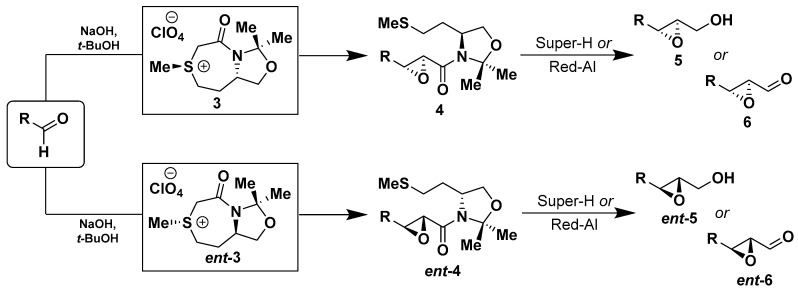
A new methodology of asymmetric epoxidation based on chiral sulfonium salts **3** and *ent*-**3**.

In connection with the Nicolaou methodology, we have recently described the design, synthesis, and reactivity of a new class of chiral sulfur ylides derived from α-amino acids, structurally characterized by the presence of a bicyclic system [62,63,64]. The new sulfonium reagents provided excellent results in the stereoselective formation of the corresponding glycidic amides. In particular, sulfonium salts **3** and *ent*-**3** reacted with simple aldehydes to obtain epoxy amides **4** and *ent*-**4**, respectively. The epoxy amides can be readily transformed into their corresponding epoxy alcohols (**5** and *ent*-**5**) or epoxy aldehydes (**6** and *ent*-**6**) (Scheme 2).

**Scheme 3:**
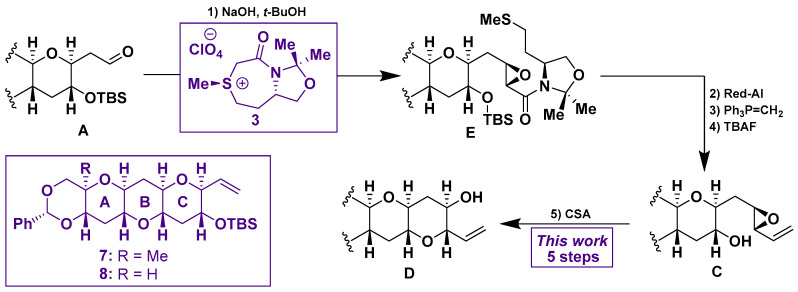
Synthetic strategy for the construction of polytetrahydropyran systems via chiral sulfonium salts **3** and *ent*-**3** and its applications in the synthesis of compounds **7** and **8**.

Having secured the synthetic efficiency that these reagents possess in the stereoselective construction of oxirane rings, the next step in our investigation was to broaden their usefulness and applicability in reactions with more complex aldehydes in order to determine the scope and limitations of this methodology. This aimed to address more challenging objectives, such as the synthesis of natural products of biological interest. In fact, the synthesis of a wide variety of natural products [65,66,67,68,69] and analogs [70,71] was efficiently prepared according to this new methodology. In our continuous efforts to exploit the synthetic value of these reagents, we decided to engage in a synthetic project directed towards the establishment of an expeditious and rapid methodology for the construction of fused polytetrahydropyran systems in an iterative manner. Thus, alternatively to the Nicolaou methodology, the methodology based on sulfonium salt **3** should reach the same synthetic precursor **C** in four steps through epoxy amide **E**. After cyclization of epoxy olefin **C** to pyran **D**, the construction of a second ring would be achieved in an iterative way containing eight steps to obtain a new tetrahydropyran (THP) system (Scheme 3). 

In light of this synthetic scheme, in the present article, we wish to report the synthesis of compounds **7** and **8**, which represent the **A**–**C** system of yessotoxin and adriatoxin and a model system lacking the angular methyl group, respectively, as suitable examples to validate the efficiency of this alternative strategy.

## 2. Results

**Scheme 4:**
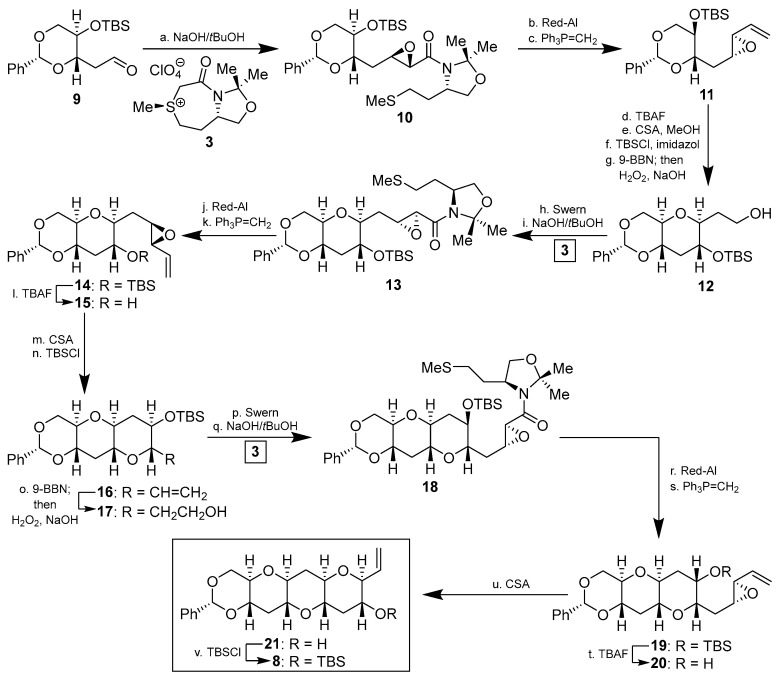
Synthesis of the *tris*-tetrahydropyranyl derivative **8**. Reagents and conditions: (a) 1.2 equiv **3**, 1.2 equiv 3.0 M aq. NaOH, CH_2_Cl_2_/H_2_O, 25 °C, 12 h, 75% (diastereoselectivity > 98%); (b) 0.5 equiv Red-Al, THF, 0 °C, 0.5 h; (c) 2.0 equiv Ph_3_PCH_3_Br, 2.0 equiv NaHMDS, THF, 0 °C, 15 min; then addition of 1.0 equiv of crude aldehyde, THF, 0 °C, 0.5 h, 81% over 2 steps; (d–g) See ref. [73]; (h) 2.5 equiv (COCl)_2_, 5.0 equiv DMSO, 7.5 equiv NEt_3_, CH_2_Cl_2_, −78 °C, 40 min; (i) 1.1 equiv **3**, 1.1 equiv 3.0 M aq. NaOH, *t*-BuOH, 25 °C, 12 h, 70% over 2 steps (diastereoselectivity > 98%); (j) 0.5 equiv Red-Al, THF, 0 °C, 0.5 h; (k) 2.0 equiv Ph_3_PCH_3_Br, 2.0 equiv NaHMDS, THF, 0 °C, 15 min; then the addition of 1.0 equiv of crude aldehyde, THF, 0 °C, 0.5 h, 77% over 2 steps; (l) 1.2 equiv TBAF, THF, 0 °C, 0.5 h, 86%; (m) 0.3 equiv CSA, CH_2_Cl_2_, 0 °C, 1 h; (n) 1.2 equiv TBSCl, 1.5 equiv imidazole, DMF, 0 °C → 25 °C, 8 h, 95% for 2 steps; (o) 1.5 equiv 9-BBN, THF, 25 °C, 1 h; then 2.25 equiv 3 M aq. NaOH, 1.65 equiv 30% H_2_O_2_, 0 °C → 25 °C, 93%; (p,q) as described in steps (h,i), 82% over 2 steps (diastereoselectivity > 98%); (r,s) as described in steps j-k, 65% over 2 steps; (t–v) as described before for steps (l–n), 74% over 3 steps. 9-BBN = 9-borabicyclo [3.3.1]nonane, CSA = (1*S*)-(+)-10-camphorsulfonic acid, Red-Al = sodium bis(2-methoxyethoxy)aluminum hydride, TBSCl = *tert*-butyldimethylsilyl chloride, TBAF = tetrabutylammonium fluoride.

To initiate this synthetic task, we decided to prepare the tricyclic system contained in the model compound **8** prior to the preparation of the **A**–**C** system of yessotoxin. To this aim, the readily available aldehyde **9** [72] was reacted with sulfonium salt **3** to afford epoxy amide **10** in 75% yield and excellent diastereoselectivity, estimated to be superior to 98% according to NMR and GC-MS spectra [64]. As we have previously demonstrated, these epoxy amides can be directly reduced to the aldehyde by the action of Red-Al [68,69]. In the case of **10**, its reaction with Red-Al at 0 °C provided a crude aldehyde, which was subjected to the treatment with the in situ prepared ylide methylene triphenylphosphorane (Ph_3_P=CH_2_) to obtain epoxy olefin **11** in an 81% overall yield from epoxy amide **10**. Epoxy olefin **11** was then prepared to be cyclized to the first tetrahydropyran ring by treatment with 10-camphorsulfonic acid (CSA), after desilylation with TBAF. The sequence of the resulting tetrahydropyranyl derivative to the alcohol **12** is described in the literature [73], entailing silylation with TBSCl and hydroboration of the double bond. After the synthesis of the first tetrahydropyran system, via the newly developed methodology, the overall yield from aldehyde **9** to compound **12** was comparable to that obtained using the Nicolaou strategy (~52%), with the added advantage of requiring fewer synthetic steps. Having prepared alcohol **12**, a second round leading to the construction of a second THP ring was commenced with a Swern oxidation, followed by reaction with sulfonium salt **3** of the resulting crude aldehyde. The result of this reaction was the obtention of epoxy amide **13** in a good 70% yield, with similar stereoselectivity (>98%) to that observed for the synthesis of **10**. From epoxy amide **13**, a synthetic sequence similar to that used for the synthesis of **12** was undertaken to obtain compound **17**, through epoxy olefins **14** and **15** and *bis*-tetrahydropyran **16**, with no issues. Finally, a third round of reactions from alcohol **17** was repeated, highlighting the reaction of the resulting aldehyde derived from alcohol **17** with sulfonium salt **3**, which again provided an excellent result in the formation of epoxy amide **18** in 82% yield and diastereoselectivity greater than 98%. The efficient transformation of **18** into the *tris*-tetrahydropyranyl derivative **8** (Scheme 4) proved the validity of this synthetic strategy which, supported in the Nicolaou methodology for the 6-*endo* cyclization reactions of hydroxyl epoxy olefins, offers a rapid, efficient, and mild stereoselective construction of *trans*-fused tetrahydropyranyl systems.

**Scheme 5:**
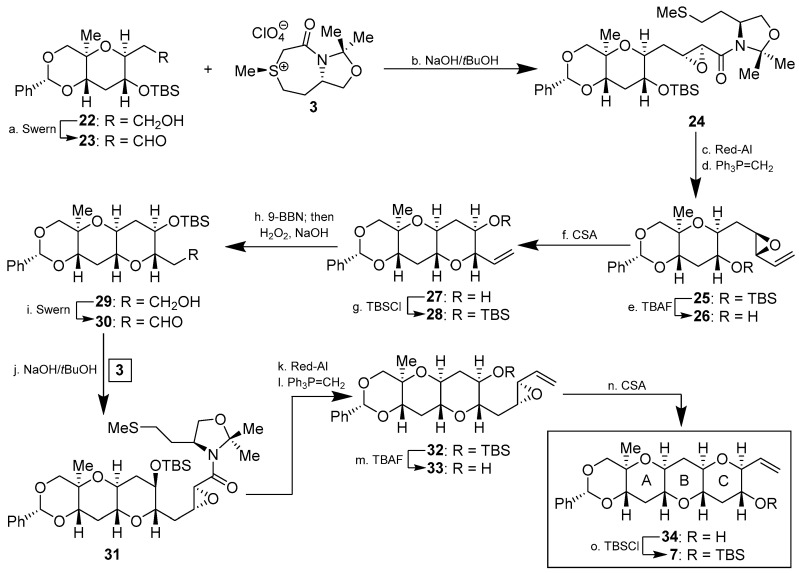
Synthesis of the **A–C** ring system **7**. Reagents and conditions: (a) 2.5 equiv (COCl)_2_, 5.0 equiv DMSO, 7.5 equiv NEt_3_, CH_2_Cl_2_, −78 °C, 40 min; (b) 1.2 equiv **3**, 1.2 equiv 3.0 M aq. NaOH, CH_2_Cl_2_/H_2_O, 25 °C, 12 h, 86% (diastereoselectivity > 98%); (c) 0.5 equiv Red-Al, THF, 0 °C, 0.5 h; (d) 2.0 equiv Ph_3_PCH_3_Br, 2.0 equiv NaHMDS, THF, 0 °C, 15 min; then addition of 1.0 equiv of crude aldehyde, THF, 0 °C, 0.5 h, 77% over 2 steps; (e) 1.2 equiv TBAF, THF, 0 °C, 0.5 h, 86%; (f) 0.3 equiv CSA, CH_2_Cl_2_, 0 °C, 1 h; (g) 1.2 equiv TBSCl, 1.5 equiv imidazole, DMF, 0 °C → 25 °C, 8 h, 95% for 2 steps; (h) 1.5 equiv 9-BBN, THF, 25 °C, 1 h; then 2.25 equiv 3 M aq. NaOH, 1.65 equiv 30% H_2_O_2_, 0 °C → 25 °C, 92%; (i) 2.5 equiv (COCl)_2_, 5.0 equiv DMSO, 7.5 equiv NEt_3_, CH_2_Cl_2_, −78 °C, 40 min; (j) 1.1 equiv **3**, 1.1 equiv 3.0 M aq. NaOH, *t*-BuOH, 25 °C, 12 h, 68% over 2 steps (diastereoselectivity > 98%); (k) 0.5 equiv Red-Al, THF, 0 °C, 0.5 h; (l) 2.0 equiv Ph_3_PCH_3_Br, 2.0 equiv NaHMDS, THF, 0 °C, 15 min; then addition of 1.0 equiv of crude aldehyde, THF, 0 °C, 0.5 h, 72% over 2 steps; (m–o) as described in steps (e–g), 69% over 3 steps.

Having demonstrated the utility of chiral sulfonium salt **3** for the iterative construction of a polytetrahydropyran system, we then extended this methodology to the synthesis of *tris*-tetrahydropyranyl derivative **7**. For this purpose, we started from the known alcohol **23** [74] that introduces a methyl group in the A ring of yessotoxin, adriatoxin, and many other marine ladder polyether-type natural products. Alcohol **22** was oxidized and the resulting aldehyde **23** reacted with sulfonium salt **3** under mild basic conditions to smoothly furnish epoxy amide **24** in excellent yield (86%) and stereoselectivity (>98%). The subsequent elaboration of **24** towards the construction of a second tetrahydropyran ring (steps c-g) is summarized in Scheme 5, being achieved in a similar sequence as described before for **16** without any problem and in very good yields. The resulting compound **28** was then transformed into its corresponding aldehyde **30**, by means of a hydroboration with 9-BBN (92%), followed by Swern oxidation, and then reacted with sulfonium salt **3** to obtain epoxy amide **31** in similar yield (68% over two steps) and stereoselectivity compared with previous cases described herein. Finally, the construction of the third ring was accomplished without difficulties in five steps to reach compound **7** on a multi-gram scale and in 50% overall yield from **31**, which could allow the continuation of the construction of a new six-membered ring (Scheme 5).

In an attempt to optimize the iterative synthetic process towards fused polytetrahydropyran systems mediated by sulfonium ylides, we explored the synthesis of the key epoxy amides by utilizing non-chiral sulfonium salts. Although the starting aldehydes do not present chirality at the alpha position to induce a stereoselective generation of the oxirane ring, we surmised that the presence of the bulky bicyclic framework close to the carbonyl group may induce a preferred conformation of the aldehyde, enough to induce a stereoselective attack of a nucleophile. As a significant precedent, the epoxidation of the allylic alcohol **35** by treatment with *m*-chloroperbenzoic acid (*m*-CPBA) proceeded in a high stereoselective fashion [75] in the formation of epoxy alcohol **36**.

**Scheme 6:**
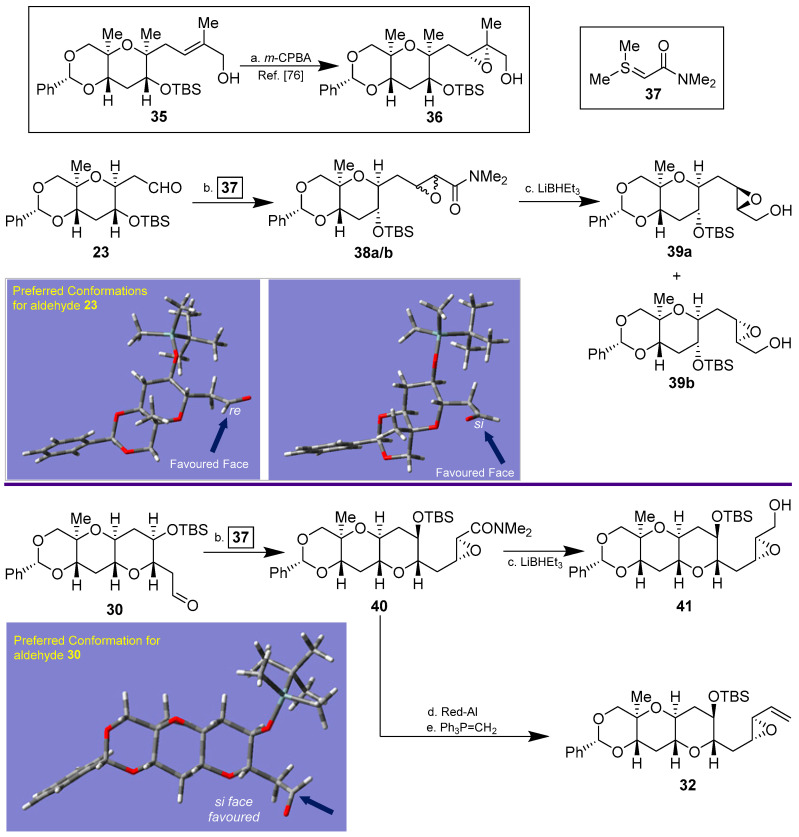
Synthesis of epoxy amides via sulfonium ylide **37** and preferred conformation of aldehydes **23** and **30**. Reagents and conditions: (a) 1.2 equiv *m*-CPBA, CH_2_Cl_2_, 0 °C, 0.5 h, 97% [75]; (b) 1.2 equiv **37**, 0 °C, 12 h, 75% for **38a/b**, 82% for **40**; (c) 2.0 equiv LiBHEt_3_, THF, 0 °C, 0.5 h, 92% for **39a:39b** (60:40), 95% for **41**. (d) 0.5 equiv Red-Al, THF, 0 °C, 0.5 h; (e) 2.0 equiv Ph_3_PCH_3_Br, 2.0 equiv NaHMDS, THF, 0 °C, 15 min; then addition of 1.0 equiv of crude aldehyde, THF, 0 °C, 0.5 h, 76% over 2 steps.

Encouraged by this result, we extended these epoxidation reactions to non-chiral sulfonium salts. In the event, when aldehyde **23** was treated with sulfonium ylide **37** [76], epoxy amides **38a/b** were obtained in a good 75% combined yield but with poor stereoselectivity, estimated in a 60:40 ratio of diastereoisomers and confirmed by reduction with lithium triethylborohydride [77] of the inseparable mixture of epoxy amides **38a/b** into the known epoxy alcohols **39a/b**. In a similar way, we explored the stereoselectivity of the more bulky aldehyde **30** with sulfonium ylide **37** to obtain in this case epoxy amide **40** as a single diastereoisomer and confirmed by its reduction into epoxy alcohol **41** (Scheme 6). Finally, epoxy amide **40** was converted into epoxy olefin **32** in a similar efficient way as for epoxy amide **31** described in Scheme 5. This interesting result of stereoselectivity opens the door towards the use of non-chiral sulfonium salts [78,79] for the stereoselective construction of epoxy amides starting from more complex aldehydes, and that remarkably facilitates the access to the key epoxide precursors of the tetrahydropyran systems. In order to rationalize the stereochemical outcome observed for the epoxidation reactions of aldehydes **23** and **30** with sulfonium ylide **37**, we undertook theoretical studies for both aldehydes. These studies consisted of a relaxed scan (using the default parameters) of the potential energy surfaces resulting from the variation of the two dihedrals present in the aldehyde side chains (O=CH-CH_2_-CH and OHC-CH_2_-CH-O) of **23** and **30** by means of the PM3 semiempirical method found in Gaussian’09. The study revealed very different results for each aldehyde, which were consistent with the observed stereochemical outcome. In particular, for aldehyde **23**, this theoretical study identified seven conformations with minimum energies, of which four displayed the *si* face as the most favored for the nucleophilic attack. In contrast, two of them showed the *re* face as the most favored for the nucleophilic attack, and one conformer presented both faces of the carbonyl group as very hindered for the nucleophilic addition (see Supporting Information). This theoretical conformational result would be in accordance with the lack of stereoselectivity found in the reaction of **23** with **37**, which delivered a 60:40 mixture of epoxy amides **38a/b**, with epoxy amide **38a** as the major stereoisomer. In contrast to aldehyde **23**, the same conformational analysis for aldehyde **30** provided only one preferred conformation with the minimum energy, detected in the dihedral-dihedral scan surface, displaying a clear preference for the *si* face for the nucleophilic approach (see Supporting Information). This theoretical result is similarly in agreement with the experimental result obtained in this case as described in Scheme 6 and should support the excellent stereoselectivity found in this reaction.

**Scheme 7:**
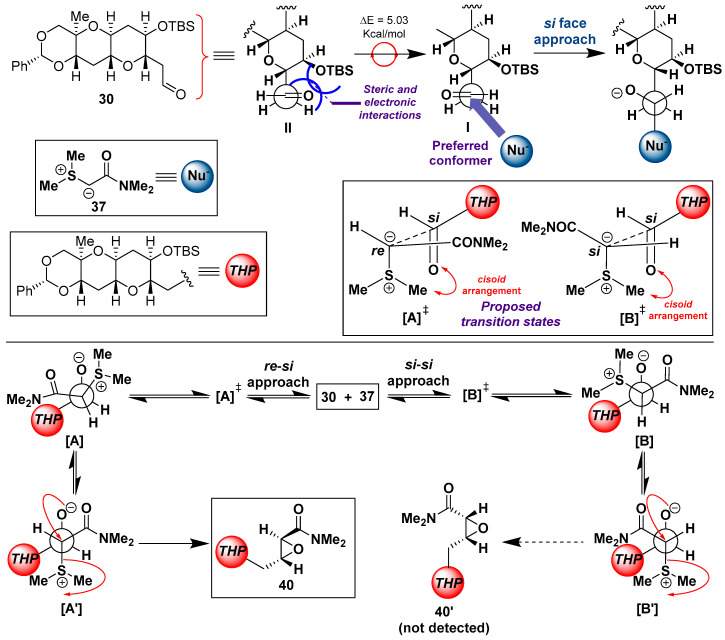
Rationale of the stereoselectivity of the reaction of aldehyde **30** with sulfonium ylide **37**.

In order to gain insight into the stereochemical outcome for the reaction of aldehyde **30** with sulfonium ylide **37**, we based on the conformer with minimum energy obtained by the previously described theoretical studies (conformer **I**), as depicted in Scheme 7. Thus, according to the Felkin–Anh model [80,81], the aldehyde adopts a conformation with the bulky alkyl group perpendicular to the carbonyl group, being the conformers **I** and **II** corresponding to the Felkin–Anh conformers and preferred for the nucleophilic attack. However, whereas the conformer **I** presented a minimum energy and, in consequence, represents the preferred conformer, the other Felkin–Anh conformer (**II**) displayed a higher energy, probably due to steric and electronic interactions between the carbonyl group and the bulky silylether.

Therefore, these results suggested that the most favorable pathway corresponds to the approach of the sulfonium ylide to the less hindered face (*si* face) of the preferred conformer of aldehyde **30**. In this nucleophilic attack two possible approaches of the sulfonium ylide should be possible, a *re*-*si* and a *si*-*si* approach corresponding to the transition states **[A]^≠^** and **[B]^≠^**. Epoxidations of carbonyl compounds via sulfonium ylides are believed to proceed via a two-step mechanism, through a preferred *cisoid* arrangement between the reactants, in which the two polar groups are in an almost eclipsed orientation [82]. According to this scenario, the kinetically favored intermediate *syn*-betaine intermediate **[A]** would be formed from the transition state **[A]^≠^**, and the subsequent step should involve rotation around the C-C bond to give betaine **[A′]**, in which the alkoxy and sulfonium groups are in an antiperiplanar conformation, prior to the formation of the oxirane ring to deliver *trans*-epoxy amide **40**. In contrast, a *re*-*si* approach, through transition state **[B]^≠^**, should deliver *cis* epoxy amide **40′**, via intermediates **[B]** and **[B′]**, which was not detected. In this case, the high energetic barrier for ring closure (**[B′] → 40′**) establishes this process as the rate-determining step and, taking into account that the betaine formation is reversible, as demonstrated in crossover experiments [82], makes this pathway nonproductive, instead reverting back to the starting aldehyde and ylide (Scheme 7). This proposed mechanism was supported by the calculation of the structures for the **[A]^≠^** and **[B]^≠^** using the same level of theory employed for the conformational analysis, which showed a difference of 3.42 Kcal/mol in favor of **[A]^≠^** and qualitatively explained the obtained experimental result (See Supporting Information).

## 3. Conclusions

As a new application of the versatile and useful chiral sulfonium salt **3**, in this article we demonstrate the validity of this reagent in a rapid and efficient preparation of *trans* fused-polycyclic ethers combining the stereoselective construction of epoxides with the Nicolaou methodology of tetrahydropyran systems via cyclization of the hydroxy epoxy olefins. This alternative strategy to produce fused tetrahydropyrans amounts to the advantage of shortening the number of steps in a linear synthesis of this class of cyclic systems compared with the classic Wittig/Sharpless asymmetric epoxidation (SAE) sequences. Furthermore, the mild and smooth reaction conditions that feature the reactions of aldehydes with sulfonium salt **3** provide an additional advantage that bodes well for its application to the rapid and efficient construction of a variety of fragments that typically occur in this class of marine toxins. In contrast to such advantages, the disadvantage that represents the necessity of using stochiometric amounts of sulfonium salt **3** is counteracted by its ease as well as its short and high-yielding synthesis on a multi-gram scale. On the other hand, the unexpected stereoselectivity found in the epoxidation of the bulky aldehyde **30** with a non-chiral sulfonium ylide, compound **37**, represents an alternative to the use of the more complex chiral sulfonium salts, such as **3**, and opens the door towards a straightforward and efficient synthesis of tetrahydropyran systems.

## 4. Experimental Section

### 4.1. General Techniques

All reactions were carried out under an argon atmosphere with dry, freshly distilled solvents under anhydrous conditions, unless using aqueous reagents or otherwise noted. All solvents used in reactions were dried and distilled using standard procedures. Tetrahydrofuran (THF) was distilled from sodium benzophenone, and methylene chloride (CH_2_Cl_2_) from calcium hydride. Yields refer to chromatographically and spectroscopically (^1^H NMR) homogeneous materials, unless otherwise stated. All solutions used in workup procedures were saturated unless otherwise noted. All reagents were purchased at the highest commercial quality and used without further purification unless otherwise stated. All reactions were monitored by thin-layer chromatography (TLC) using 0.25 mm silica gel plates (60F-254) using UV light (254 nm) as a visualizing agent and acidic ceric ammonium molybdate/phosphomolybdic acid or potassium permanganate solutions and heat as developing agents. Flash column chromatography (FCC) was performed using silica gel (60 Å, particle size 230–400 mesh) under air pressure. All solvents used for chromatographic purifications were distilled prior to use. ^1^H and ^13^C NMR spectra were recorded on a Bruker DPX-400 MHz instrument (Fällanden, Switzerland) and calibrated using residual undeuterated solvent as an internal reference. Chemical shifts are reported in ppm, with the resonance resulting from incomplete deuteration of the solvent as the internal standard (^13^CDCl_3_: 7.26 ppm, s and 77.0 ppm, t; ^13^CD_3_OD: 4.87 ppm, s, 3.31 ppm, quin and 49.1 ppm, sep; ^13^C_2_D_6_OS: 2.49 ppm, quin and 39.52 ppm, sep). Data are reported as follows: chemical shift δ/ppm (multiplicity, coupling constants *J* (Hz) and integration (^1^H only)). The following abbreviations were used to explain the multiplicities: s = singlet; d = doublet; t = triplet; q = quartet; quin = quintet; b = broad; and m = multiplet or combination thereof. ^13^C signals are singles unless otherwise stated. High-resolution mass spectrometry (HRMS) was performed on a Thermo Fisher Scientific H-ESI and APCI mass spectrometer (Waltham (MA), USA) in positive mode and using an ion trap (Orbitrap) as the mass analyzer type. HRMS signals are reported to four decimal places and are within ±5 ppm of theoretical values. Theoretical calculations were performed with the Gaussian’09 package using PM3 in vacuo as the calculation method for the relaxed PES scan [83].

### 4.2. Synthesis of the A-C System Precursor 7

**Epoxy Amide 10.** To a suspension of aldehyde **9** (2.00 g, 5.90 mmol, 1.0 equiv) and sulfonium salt **3** (2.25 g, 7.1 mmol, 1.2 equiv) in CH_2_Cl_2_ (50 mL) was added a 3.0 M aqueous NaOH solution (1.4 mL, 7.1 mmol, 1.2 equiv) at 25 °C. After 12 h at this temperature, the crude mixture was diluted with Et_2_O, washed with saturated aqueous NH_4_Cl solution, and, after decantation, the aqueous phase was extracted twice with Et_2_O. The combined organic extracts were washed with brine, dried over MgSO_4_, filtered, and the solvent evaporated under reduced pressure. The crude product was purified by flash column chromatography (silica gel, 20% EtOAc in hexanes) to obtain the epoxy amide **10** (2.34 g, 75%) as a yellow oil: R*_f_* = 0.36 (silica gel, 20% EtOAc in hexanes); [α]^20^_D_ = –16.3 (*c* 2.8, CH_2_Cl_2_); ^1^H NMR (400 MHz, CDCl_3_): *δ* = 0.04 (s, 3 H), 0.06 (s, 3 H), 0.82 (s, 9 H), 1.46 (s, 3 H), 1.57 (s, 3 H), 1.85–1.76 (m, 1 H), 1.92 (ddd, *J* = 14.6, 6.2, 5.6 Hz, 1 H), 2.00 (s, 3 H), 2.11 (dd, *J* = 6.9, 3.3 Hz, 1 H), 2.16 (dd, *J* = 4.2, 3.4 Hz, 1 H), 2.25–2.19 (m, 1 H), 2.28 (dd, *J* = 7.4, 5.0 Hz, 1 H), 3.41–3.34 (m, 2 H), 3.51 (dd, *J* = 10.0, 9.9 Hz, 1 H), 3.72–3.62 (m, 2 H), 3.74 (d, *J* = 9.2 Hz, 1 H), 3.87 (dd, *J* = 9.2, 4.9 Hz, 1 H), 4.10–4.05 (m, 1 H), 4.13 (dd, *J* = 10.9, 4.8 Hz, 1 H), 5.44 (s, 1 H), 7.32–7.20 (m, 3 H), 7.42–7.39 (m, 2 H); ^13^C NMR (100 MHz, CDCl_3_): *δ* = −4.9, −4.3, 15.7, 17.7, 22.8, 25.6, 26.2, 30.4, 32.6, 34.2, 53.2, 55.2, 55.7, 65.4, 66.8, 71.7, 79.3, 95.7, 101.1, 126.3, 128.2, 128.9, 165.7; HRMS (ESI-TOF) *m*/*e* 552.2825, M + H^+^ calcd. for C_28_H_46_NO_6_SSi 552.2815.

**Epoxy Alkene 11.** To a solution of epoxy amide **10** (2.00 g, 3.62 mmol, 1.0 equiv) in THF (30 mL) was added dropwise Red-Al (0.51 mL, 70% *w*/*v* in toluene, 1.81 mmol, 0.5 equiv) at 0 °C. After 30 min at this temperature, the reaction mixture was quenched by the addition of a saturated aqueous Na^+^/K^+^ tartrate solution and diluted with EtOAc. The resulting mixture was vigorously stirred until a clear separation of both organic and aqueous phases. After separation of both layers, the aqueous phase was extracted with EtOAc and the combined organic extracts were washed with H_2_O and brine, dried over anhydrous MgSO_4_, and the solvent evaporated under reduced pressure. The resulting crude aldehyde was used in the next step without further purification. To a stirred suspension of methyl triphenylphosphonium bromide (2.58 g, 7.24 mmol, 2.0 equiv) in THF (20 mL) at 0 °C was added dropwise NaHMDS (7.24 mL, 1.0 M in THF, 7.24 mmol, 2.0 equiv). The resulting suspension was stirred for 15 min at this temperature, and after this time, a solution of the crude aldehyde (~3.62 mmol) in THF (30 mL) was added dropwise, and the resulting mixture was stirred for 30 min. After this time, the crude mixture was diluted with Et_2_O and washed with saturated aqueous NH_4_Cl solution. The aqueous phase was extracted with Et_2_O and the combined organic extracts were washed with brine, dried over MgSO_4_, filtered, and the solvent evaporated under reduced pressure. The crude product was purified by flash column chromatography (silica gel, 20% EtOAc in hexanes) to obtain the epoxy alkene **11** (1.10 g, 81% over two steps) as a colorless oil whose spectroscopic and physical properties matched with those described in the literature [73].

**Epoxy Amide 13.** (COCl)_2_ (1.03 mL, 11.73 mmol, 2.5 equiv) was dissolved in CH_2_Cl_2_ (20 mL) and cooled at −78 °C. DMSO (1.7 mL, 23.46 mmol, 5.0 equiv) was then added dropwise and the mixture was stirred for 10 min at the same temperature. Then, a solution of alcohol **12** (1.81 g, 4.69 mmol, 1.0 equiv) in CH_2_Cl_2_ (10 mL) was added slowly, and the reaction mixture was stirred for 40 min at −78 °C then quenched by addition of Et_3_N (4.9 mL, 35.18 mmol, 7.5 equiv). The resulting mixture was warmed to 25 °C and stirred for 1 h. After this time, the crude mixture was diluted with Et_2_O and washed with saturated aqueous NH_4_Cl solution. After the separation of both layers, the organic phase was washed with water and brine, dried over MgSO_4_, and the solvent evaporated under reduced pressure to afford the crude aldehyde that was used in the next step without further purification. To a solution of sulfonium salt **3** (1.63 g, 5.16 mmol, 1.1 equiv) in *t*BuOH (20 mL) was added a 3.0 M aqueous NaOH solution (1.72 mL, 5.16 mmol, 1.1 equiv) at 25 °C. After 15 min at this temperature, a solution of the crude aldehyde (~4.69 mmol, 1.0 equiv) in *t*BuOH (10 mL) was added and the reaction mixture was stirred for 12 h at 25 °C. After this time, the crude mixture was diluted with EtOAc and the organic layer was washed sequentially with H_2_O and brine, dried over MgSO_4_, filtered, and the solvent evaporated under reduced pressure. The crude product was purified by flash column chromatography (silica gel, 20% EtOAc in hexanes) to obtain the epoxy amide **13** (1.98 g, 70% over two steps) as a colorless oil: R*_f_* = 0.42 (silica gel, 20% EtOAc in hexanes); [α]^20^_D_ = –10.5 (*c* 1.5, CH_2_Cl_2_); ^1^H NMR (400 MHz, CDCl_3_) *δ* = 0.02 (s, 3 H), 0.03 (s, 3 H), 0.80 (s, 9 H), 1.19 (dd, *J* = 9.0, 8.8 Hz, 1 H), 1.48 (s, 3 H), 1.59 (s, 3 H), 1.74–1.64 (m, 2 H), 2.04–1.98 (m, 1 H), 2.06 (s, 3 H), 2.13 (ddd, *J* = 14.6, 3.8 Hz, 1 H), 2.37–2.32 (m, 1 H), 2.44–2.39 (m, 1 H), 2.54 (ddd, *J* = 12.8, 7.4, 5.2 Hz, 1 H), 3.27 (d, *J* = 1.8 Hz, 1 H), 3.40–3.31 (m, 3 H), 3.49 (ddd, *J* = 12.2, 9.1, 4.0 Hz, 1 H), 3.66–3.58 (m, 2 H), 3.84 (d, *J* = 9.1 Hz, 1 H), 3.97 (dd, *J* = 9.0, 5.1 Hz, 1 H), 4.08–4.03 (m, 1 H), 4.24 (dd, *J* = 10.4, 4.9 Hz, 1 H), 5.45 (s, 1 H), 7.32–7.28 (m, 3 H), 7.43–7.41 (m, 2 H); ^13^C NMR (100 MHz, CDCl_3_) *δ* = −4.9, −4.2, 15.9, 17.8, 22.8, 25.6, 26.2, 30.7, 32.9, 34.4, 38.7, 52.8, 55.4, 55.7, 66.8, 69.1, 69.2, 73.0, 76.2, 80.1, 95.8, 101.6, 125.6, 126.0, 128.2, 128.9, 137.2, 163.9; HRMS (ESI-TOF) *m*/*e* 608.3102, M + H^+^ calcd. for C_31_H_49_NO_7_SSi 608.3077.

**Epoxy Alkene 14.** Epoxy amide **13** (1.76 g, 2.90 mmol, 1.0 equiv) was treated with Red-Al (0.41 mL, 70% *w*/*v* in toluene, 1.45 mmol, 0.5 equiv) and the resulting aldehyde was treated with freshly prepared methylentriphenilphosphorane (5.80 mmol, 2.0 equiv) in exactly the same manner as previously described for synthesis of **11** to afford epoxy alkene **14** (970 mg, 77% over two steps) as a white foam: R*_f_* = 0.53 (silica gel, 40% EtOAc in hexanes); [α]^20^_D_ = –14.8 (*c* 1.0, CH_2_Cl_2_); ^1^H NMR (400 MHz, CDCl_3_) *δ* = 0.02 (s, 3 H), 0.04 (s, 3 H), 0.81 (s, 9 H), 1.72–1.60 (m, 2 H), 1.97 (ddd, *J* = 14.4, 5.1, 3.1 Hz, 1 H), 2.34 (ddd, *J* = 11.6, 4.3, 4.1 Hz, 1 H), 2.99 (dt, *J* = 5.6, 2.1 Hz, 1 H), 3.03 (dd, *J* =7.4, 2.1 Hz, 1 H), 3.35–3.29 (m, 2 H), 3.56–3.45 (m, 2 H), 3.62 (dd, *J* = 10.3, 10.1 Hz, 1 H), 4.26 (dd, *J* = 10.5, 5.0 Hz, 1 H), 5.23 (dd, *J* = 10.0, 1.7 Hz, 1 H), 5.42 (dd, *J* = 17.2, 1.7 Hz, 1 H), 5.46 (s, 1 H), 5.53 (ddd, *J* = 17.3, 10.2, 7.5 Hz, 1 H), 7.31–7.29 (m, 3 H), 7.44–7.42 (m, 2 H); ^13^C NMR (100 MHz, CDCl_3_) *δ* = −4.9, −4.2, 25.7, 26.2, 30.9, 37.7, 63.8, 65.9, 68.2, 76.7, 78.2, 89.1, 101.7, 121,6, 125.6, 126.0, 128.2, 128.9, 135.2; HRMS (ESI-TOF) *m*/*e* 433.2398, M + H^+^ calcd. for C_24_H_36_O_5_Si 433.2410.

**Alcohol 15.** To a solution of silyl ether **14** (850 mg, 1.96 mmol, 1.0 equiv) in THF (15 mL) was added TBAF (2.35 mL, 1.0 M in THF, 2.35 mmol, 1.2 equiv) at 0 °C. After 30 min, the reaction mixture was diluted with Et_2_O and washed with a saturated aqueous NH_4_Cl solution. After separation of both layers, the aqueous phase was then extracted with Et_2_O and the combined organic extracts were washed with brine, dried over MgSO_4_, filtered, and the solvent evaporated under reduced pressure. The crude product was purified by flash column chromatography (silica gel, 50% EtOAc in hexanes) to afford alcohol **15** (600 mg, 96%) as a white foam.

**Silyl Ether 16.** Alcohol **15** (500 mg, 1.57 mmol, 1.0 equiv) was dissolved in CH_2_Cl_2_ (10 mL) and the solution was treated with (1*S*)-(+)-10-CSA (109 mg, 0.47 mmol, 0.3 equiv) at 0 °C. After 1 h at this temperature, Et_3_N (0.11 mL, 0.79 mmol, 0.5 equiv) was added and the mixture was stirred for 30 min. After this time, the solvents were evaporated under reduced pressure and the resulting crude product was purified by flash column chromatography (silica gel, 50% EtOAc in hexanes) to afford the corresponding *bis*-pyran derivative. To a solution of the hidroxyl *bis*-pyran (380 mg, 1.19 mmol, 1.0 equiv) in DMF (10 mL) was added imidazole (121 mg, 1.78 mmol, 1.5 equiv) and TBSCl (215 mg, 1.43 mmol, 1.2 equiv) at 0 °C. After 8 h at 25 °C, the mixture was quenched with MeOH, diluted with Et_2_O, and washed with a saturated aqueous NH_4_Cl solution. After separation of both layers, the aqueous phase was then extracted with Et_2_O, and the combined organic extracts were washed with H_2_O and brine, dried over MgSO_4_, filtered, and the solvent evaporated under reduced pressure. The crude product was purified by flash column chromatography (silica gel, 10% EtOAc in hexanes) to afford silyl ether **16** (490 mg, 95%) as a yellow oil: R*_f_* = 0.35 (silica gel, 50% EtOAc in hexanes); [α]^20^_D_ = –25.9 (*c* 1.2, CH_2_Cl_2_); ^1^H NMR (400 MHz, CDCl_3_) *δ* = 0.02 (s, 3 H), 0.04 (s, 3 H), 0.85 (s, 9 H), 1.75–1.64 (m, 2 H), 2.29 (ddd, *J* = 14.4, 5.1, 3.1 Hz, 1 H), 2.34 (ddd, *J* = 11.6, 4.3, 4.1 Hz, 1 H), 3.35 (dt, *J* = 5.6, 2.1 Hz, 1 H), 3.58–3.50 (m, 3 H), 3.63–3.60 (m, 2 H), 3.75 (dd, *J* = 10.3, 9.9 Hz, 1 H), 4.35 (dd, *J* = 10.5, 5.0 Hz, 1 H), 5.27 (dd, *J* = 10.0, 1.7 Hz, 1 H), 5.38 (dd, *J* = 17.2, 1.7 Hz, 1 H), 5.51 (s, 1 H), 5.85 (ddd, *J* = 17.3, 10.2, 7.5 Hz, 1 H), 7.30–7.28 (m, 3 H), 7.42–7.39 (m, 2 H); ^13^C NMR (100 MHz, CDCl_3_) *δ* = −4.9, −4.2, 18.9, 25.6, 26.2, 35.4, 39.7, 68.9, 70.1, 73.8, 76.7, 82.5, 102.7, 117,7, 125.6, 126.0, 128.2, 128.9, 137.2, 142.9; HRMS (ESI-TOF) *m*/*e* 433.2398, M + H^+^ calcd. for C_24_H_36_O_5_Si 433.2410.

**Alcohol 17.** To a solution of alkene **16** (450 mg, 1.04 mmol, 1.0 equiv) in THF (10 mL) was added 9-BBN (3.12 mL, 0.5 M in THF, 1.56 mmol, 1.5 equiv) at 25 °C. After 1 h, the reaction mixture was cooled to 0 °C and was treated dropwise with a 3.0 N aqueous NaOH solution (0.78 mL, 2.34 mmol, 2.25 equiv) and H_2_O_2_ (0.21 mL, 30% *w*/*v*, 1.72 mmol, 1.65 equiv). The reaction mixture was warmed to 25 °C and stirred for 2 h. After this time, the crude mixture was diluted with Et_2_O and washed with H_2_O. After separation of both layers, the aqueous phase was then extracted with Et_2_O, and the combined organic extracts were washed with brine, dried over MgSO_4_, filtered, and the solvent evaporated under reduced pressure. The crude product was purified by flash column chromatography (silica gel, 20 → 50% EtOAc in hexanes) to afford alcohol **17** (436 mg, 93%) as a colorless oil: R*_f_* = 0.34 (silica gel, 50% EtOAc in hexanes); [α]^20^_D_ = −26.5 (*c* 0.8, CH_2_Cl_2_); ^1^H NMR (400 MHz, CDCl_3_) *δ* = 0.02 (s, 3 H), 0.03 (s, 3 H), 0.82 (s, 9 H), 1.51–1.42 (m, 2 H), 1.67–1.56 (m, 2 H), 2.05–1.98 (m, 1 H), 2.10 (bs, 1 H), 2.26 (ddd, *J* = 11.9, 4.1, 3.9 Hz, 1 H), 2.35 (dt, *J* = 11.5, 3.7 Hz, 1 H), 3.30–3.11 (m, 2 H), 3.37–3.28 (m, 2 H), 3.42 (ddd, *J* = 10.6, 9.0, 4.6 Hz, 1 H), 3.52 (ddd, *J* = 11.6, 9.1, 4.2 Hz, 1 H), 3.64 (dd, *J* = 10.3, 10.0 Hz, 1 H), 3.75 (dd, *J* = 5.8, 5.5 Hz, 1 H), 4.25 (dd, *J* = 10.4, 4.8 Hz, 1 H), 5.47 (s, 1 H), 7.33–7.20 (m, 3 H), 7.44–7.42 (m, 2 H); ^13^C NMR (100 MHz, CDC1_3_) *δ* = −4.9, −4.2, 17.8, 25.6, 33.3, 34.7, 38.9, 53.1, 57.7, 61.5, 69.1, 69.7, 73.4, 76.3, 80.2, 101.6, 126.0, 128.4, 128.9, 137.2; HRMS (ESI-TOF) *m*/*e* 451.2528, M + H^+^ calcd. for C_24_H_39_O_6_Si 451.2516.

**Epoxy Amide 18**. Alcohol **17** (355 mg, 0.79 mmol) was oxidized by Swern oxidation, and the corresponding aldehyde was treated with sulfonium salt **3** (298 mg, 0.95 mmol, 1.20 equiv) in the same manner as previously described for the synthesis of **13** to afford epoxy amide **18** (430 mg, 82% over two steps) as a colorless oil: R*_f_* = 0.46 (silica gel, 50% EtOAc in hexanes); [α]^20^_D_ = −22.4 (*c* 1.2, CH_2_Cl_2_); ^1^H NMR (400 MHz, CDCl_3_) *δ* = 0.02 (s, 3 H), 0.03 (s, 3 H), 0.80 (s, 9 H), 1.47 (s, 3 H), 1.54–1.51 (m, 1 H), 1.59 (s, 3 H), 1.77–1.61 (m, 4 H), 2.04–1.98 (m, 1 H), 2.06 (s, 3 H), 2.14 (dt, *J* = 14.9, 3.8 Hz, 1 H), 2.26 (dt, *J* = 11.4, 3.7 Hz, 1 H), 2.35 (dt, *J* = 11.2, 3.5 Hz, 1 H), 2.41–2.38 (m, 1 H), 2.50 (ddd, *J* = 14.5, 7.8, 5.2 Hz, 1 H), 3.14–3.11 (m, 2 H), 3.37–3.25 (m, 3 H), 3.57–3.49 (m, 2 H), 3.63 (dd, *J* = 10.3, 9.9 Hz, 1 H), 3.83 (d, *J* = 9.1 Hz, 1 H), 3.95 (dd, *J* = 9.1, 5.0 Hz, 1 H), 4.20 (ddd, *J* = 10.0, 8.2, 3.1 Hz, 1 H), 4.25 (dd, *J* = 10.4, 4.8 Hz, 1 H), 5.47 (s, 1 H), 7.33–7.28 (m, 3 H), 7.44–7.42 (m, 2 H); ^13^C NMR (100 MHz, CDC1_3_) *δ* = −4.8, −4.2, 16.0, 17.8, 22.9, 25.7, 26.3, 30.7, 32.9, 34.5, 34.8, 39.0, 53.1, 55.5, 55.9, 66.9, 69.1, 69.3, 73.4, 76.5, 77.2, 79.9, 95.8, 101.8, 126.1, 128.3, 129.1, 137.2, 164.0; HRMS (ESI-TOF) *m*/*e* 664.3327, M + H^+^ calcd. for C_34_H_54_NO_8_SSi 664.3339.

**Epoxy Alkene 19**. Epoxy amide **18** (120 mg, 0.18 mmol, 1.0 equiv) was treated with Red-Al (26 µL, 70% *w*/*v* in toluene, 0.09 mmol, 0.5 equiv) and the resulting aldehyde was treated with freshly prepared methylentriphenilphosphorane (0.36 mmol, 2.0 equiv) in exactly the same manner as previously described for synthesis of **14** to afford epoxy alkene **19** (57 mg, 65% over two steps) as a white foam: R*_f_* = 0.48 (silica gel, 20% EtOAc in hexanes); [α]^20^_D_ = –15.6 (*c* 0.4, CH_2_Cl_2_); ^1^H NMR (400 MHz, CDCl_3_) *δ* = 0.02 (s, 3 H), 0.04 (s, 3 H), 0.82 (s, 9 H), 1.47 (q, *J* = 11.2 Hz, 1 H), 1.76–1.60 (m, 2 H), 1.93 (ddd, *J* = 14.4, 5.2, 3.1 Hz, 1 H), 2.27 (dt, *J* = 11.8, 4.0 Hz, 1 H), 2.37 (dt, *J* = 11.1, 3.8 Hz, 1 H), 3.02 (dt, *J* = 5.4, 2.1 Hz, 1 H), 3.05 (dd, *J* = 7.4, 2.1 Hz, 1 H), 3.14–3.09 (m, 2 H), 3.22 (dt, *J* = 7.8, 3.0 Hz, 1 H), 3.56–3.46 (m, 2 H), 3.39–3.32 (m, 1 H), 3.64 (dd, *J* = 10.3, 10.0 Hz, 1 H), 4.25 (dd, *J* = 10.5, 4.8 Hz, 1 H), 5.21 (dd, *J* = 10.0, 1.5 Hz, 1 H), 5.41 (dd, *J* = 17.2, 1.6 Hz, 1 H), 5.53 (ddd, *J* = 17.3, 10.0, 7.4 Hz, 1 H), 5.47 (s, 1 H), 7.33–7.29 (m, 3 H), 7.45–7.43 (m, 2 H); ^13^C NMR (100 MHz, CDC1_3_) *δ* = −4.8, −4.2, 17.8, 25.7, 33.8, 34.8, 39.0, 101.7, 119.1, 126.1, 128.3, 129.0, 135.8, 137.2; HRMS (ESI-TOF) *m*/*e* 489.2648, M + H^+^ calcd. for C_27_H_41_O_6_Si 489.2672.

***Tris*-Tetrahydropyran 8.** Epoxy alkene **19** (50 mg, 0.102 mmol) was treated sequentially with TBAF, CSA and TBSCl in exactly the same manner as previously described for synthesis of **16** to afford *tris*-tetrahydropyran **8** (37 mg, 74% over three steps) as a white solid: R*_f_* = 0.58 (silica gel, 20% EtOAc in hexanes); [α]^20^_D_ = −15.8 (*c* 0.2, CH_2_Cl_2_); ^1^H NMR (400 MHz, CDCl_3_) *δ* = −0.02 (s, 3 H), 0.01 (s, 3 H), 0.82 (s, 9 H), 1.47 (q, *J* = 11.1 Hz, 1 H), 1.52 (q, *J* = 11.2 Hz, 1 H), 1.67 (q, *J* = 11.3 Hz, 1 H), 2.42–2.28 (m, 3 H), 3.19–3.05 (m, 3 H), 3.44–3.36 (m, 3 H), 3.57–3.53 (m, 2 H), 3.65 (dd, *J* = 10.3 Hz, 1 H), 4.27 (dd, *J* = 10.4, 4.8 Hz, 1 H), 5.19 (dd, *J* = 10.7, 1.0 Hz, 1 H), 5.32 (dt, *J* = 17.2, 1.4 Hz, 1 H), 5.48 (s, 1 H), 5.81 (ddd, *J* = 17.1, 10.6, 6.2 Hz, 1 H), 7.32–7.29 (m, 3 H), 7.46–7.43 (m, 2 H); ^13^C NMR (100 MHz, CDC1_3_) *δ* = −4.6, −4.3, 17.9, 25.7, 34.8, 35.0, 39.2, 69.1, 70.3, 73.6, 76.1, 76.5, 76.8, 77.0, 77.2, 83.2, 101.8, 117.9, 126.1, 128.3, 129.1, 135.7, 137.2; HRMS (ESI-TOF) *m*/*e* 489.2665, M + H^+^ calcd. for C_27_H_41_O_6_Si 489.2672.

**Epoxy Amide 24.** Aldehyde **23** (2.74 g, 6.74 mmol) was treated with sulfonium salt **3** (2.55 g, 8.09 mmol, 1.20 equiv) in exactly the same manner as previously described for synthesis of **13** to afford epoxy amide **24** (3.60 g, 86%) as a colorless oil: R*_f_* = 0.47 (silica gel, 20% EtOAc in hexanes); [α]^20^_D_ = −24.6 (*c* 1.0, CH_2_Cl_2_); ^1^H NMR (400 MHz, CDCl_3_) *δ* = 0.02 (s, 3 H), 0.03 (s, 3 H), 0.80 (s, 9 H), 1.19 (dd, *J* = 9.0, 8.8 Hz, 1 H), 1.48 (s, 3 H), 1.59 (s, 3 H), 1.74–1.64 (m, 2 H), 2.04–1.98 (m, 1 H), 2.06 (s, 3 H), 2.13 (ddd, *J* = 14.6, 3.8, 3.5 Hz, 1 H), 2.37–2.32 (m, 1 H), 2.44–2.39 (m, 1 H), 2.54 (ddd, *J* = 12.8, 7.4, 5.2 Hz, 1 H), 3.27 (d, *J* = 1.8 Hz, 1 H), 3.40–3.31 (m, 3 H), 3.49 (ddd, *J* = 12.2, 9.1, 4.0 Hz, 1 H), 3.66–3.58 (m, 2 H), 3.84 (d, *J* = 9.1 Hz, 1 H), 4.25 (dd, *J* = 9.0, 5.1 Hz, 1 H), 5.52 (s, 1 H), 7.33–7.28 (m, 3 H), 7.44–7.42 (m, 2 H); ^13^C NMR (100 MHz, CDC1_3_) *δ* = −4.9, −4.2, 15.9, 17.8, 22.8, 25.6, 26.2, 30.7, 32.9, 34.4, 38.7, 52.8, 55.4, 55.7, 66.8, 69.1, 69.2, 73.0, 76.2, 80.1, 95.8, 101.6, 125.6, 126.0, 128.2, 128.9, 137.2, 163.9; HRMS (ESI-TOF) *m*/*e* 622.3218, M + H^+^ calcd. for C_33_H_51_NO_7_SSi 622.3234.

**Epoxy Alkene 25**. Epoxy amide **24** (1.76 g, 2.90 mmol, 1.0 equiv) was treated with Red-Al (0.41 mL, 70% *w*/*v* in toluene, 1.45 mmol, 0.5 equiv) and the resulting aldehyde was treated with freshly prepared methylentriphenilphosphorane (5.80 mmol, 2.0 equiv) in exactly the same manner as previously described for synthesis of **14** to afford epoxy alkene **25** (970 mg, 77% over two steps) as a white foam: R*_f_* = 0.53 (silica gel, 40% EtOAc in hexanes); [α]^20^_D_ = −32.4 (*c* 1.2, CH_2_Cl_2_); ^1^H NMR (400 MHz, CDCl_3_) *δ* = 0.02 (s, 3 H), 0.04 (s, 3 H), 0.81 (s, 9 H), 1.72–1.60 (m, 2 H), 1.97 (ddd, *J* = 14.4, 5.1, 3.1 Hz, 1 H), 2.34 (ddd, *J* = 11.6, 4.3 Hz, 1 H), 2.99 (dt, *J* = 5.6, 2.1 Hz, 1 H), 3.03 (dd, *J* = 7.4, 2.1 Hz, 1 H), 3.35–3.29 (m, 2 H), 3.56–3.45 (m, 2 H), 3.62 (dd, *J* = 10.3 Hz, 1 H), 4.26 (dd, *J* = 10.5, 5.0 Hz, 1 H), 5.23 (dd, *J* = 10.0, 1.7 Hz, 1 H), 5.42 (dd, *J* = 17.2, 1.7 Hz, 1 H), 5.46 (s, 1 H), 5.53 (ddd, *J* = 17.3, 10.2, 7.5 Hz, 1 H), 7.31–7.29 (m, 3 H), 7.44–7.42 (m, 2 H); ^13^C NMR (100 MHz, CDCl_3_) *δ* = −4.9, −4.2, 15.9, 17.8, 22.8, 25.6, 26.2, 30.7, 32.9, 34.4, 38.7, 52.8, 55.4, 55.7, 66.8, 69.1, 69.2, 73.0, 76.2, 80.1, 95.8, 101.6, 125.6, 126.0, 128.2, 128.9, 137.2, 163.9; HRMS (ESI-TOF) *m*/*e* 447.2582, M + H^+^ calcd. for C_25_H_38_O_5_Si 447.2567.

**Alcohol 29.** Alkene **28** (2.20 g, 4.93 mmol, 1.0 equiv) was treated with 9-BBN (14.80 mL, 0.5 M in THF, 7.40 mmol, 1.5 equiv) and an oxidative work up (H_2_O_2_, NaOH) in exactly the same manner as previously described for synthesis of **17** to afford alcohol **29** (2.11 g, 92%) as a white foam: R*_f_* = 0.38 (silica gel, 50% EtOAc in hexanes); [α]^20^_D_ = −16.5 (*c* 1.2, CH_2_Cl_2_); ^1^H NMR (400 MHz, CDCl_3_) *δ* = 0.02 (s, 3 H), 0.03 (s, 3 H), 0.82 (s, 9 H), 1.43 (q, *J* = 11.3 Hz, 1 H), 1.46 (s, 3 H), 1.64–1.54 (m, 1 H), 1.73 (q, *J* = 11.1 Hz, 1 H), 2.05–1.98 (m, 1 H), 2.19–2.12 (m, 2 H), 2.29 (bs, 1 H), 3.10 (ddd, *J* = 11.3, 9.6, 4.6 Hz, 1 H), 3.29 (dt, *J* = 9.1, 2.5 Hz, 1 H), 3.47–3.36 (m, 2 H), 3.57 (dd, *J* = 6.3, 4.1 Hz, 1 H), 3.60 (bs, 1 H), 3.74 (dd, *J* = 5.3, 4.8 Hz, 2 H), 3.89 (d, *J* = 10.1 Hz, 1 H), 5.51 (s, 1 H), 7.33–7.20 (m, 3 H), 7.43–7.41 (m, 2 H); ^13^C NMR (100 MHz, CDCl_3_) *δ* = −4.9, −4.2, 15.3, 17.7, 25.7, 33.2, 34.6, 38.9, 54.1, 58.2, 61.3, 68.5, 69.8, 73.5, 76.3, 80.1, 101.6, 126.2, 128.4, 128.9, 137.3; HRMS (ESI-TOF) *m*/*e* 465.2659, M + H^+^ calcd. for C_25_H_41_O_6_Si 465.2672.

**Epoxy Amide 31.** Alcohol **29** (1.40 g, 3.01 mmol) was oxidized by Swern oxidation, and the corresponding aldehyde **30** was treated with sulfonium salt **3** (1.14 g, 3.61 mmol, 1.20 equiv) in the same manner as previously described for synthesis of **18** to afford epoxy amide **31** (1.66 g, 68% over two steps) as a colorless oil: R*_f_* = 0.38 (silica gel, 30% EtOAc in hexanes); [α]^20^_D_ = −31.6 (*c* 2.0, CH_2_Cl_2_); ^1^H NMR (400 MHz, CDCl_3_) *δ* = 0.08 (s, 3 H), 0.09 (s, 3 H), 0.84 (s, 9 H), 1.48 (q, *J* = 11.5 Hz, 1 H), 1.52 (s, 3 H), 1.55 (s, 3 H), 1.65 (s, 3 H), 1.84–1.73 (m, 3 H), 2.10–2.04 (m, 2 H), 2.12 (s, 3 H), 2.25–2.17 (m, 2 H), 2.49–2.42 (m, 1 H), 2.65–2.52 (m, 1 H), 3.16 (ddd, *J* = 11.2, 9.4, 4.6 Hz, 1 H), 3.33 (ddd, *J* = 9.4, 6.7, 3.5 Hz, 1 H), 3.36 (d, *J* = 2.0 Hz, 1 H), 3.39 (ddd, *J* = 6.2, 3.9, 2.0 Hz, 1 H), 3.46 (ddd, *J* = 11.6, 9.4, 3.9 Hz, 1 H), 3.66–3.60 (m, 3 H), 3.89 (d, *J* = 9.1 Hz, 1 H), 3.95 (d, *J* = 10.1 Hz, 1 H), 4.00 (ddd, *J* = 9.1, 5.2, 1.0 Hz, 1 H), 4.26 (ddd, *J* = 10.4, 4.8, 3.1 Hz, 1 H), 5.56 (s, 1 H), 7.40–7.34 (m, 3 H), 7.49–7.47 (m, 2 H); ^13^C NMR (100 MHz, CDC1_3_) *δ* = −4.8, −4.2, 15.0, 15.9, 17.8, 22.9, 25.7, 26.3, 30.5, 30.8, 32.9, 34.5, 39.5, 53.1, 55.5, 55.9, 66.9, 69.1, 69.6, 69.9, 76.2, 77.8, 79.7, 80.1, 95.8, 102.9, 126.2, 128.3, 129.1, 137.4, 164.0; HRMS (ESI-TOF) *m*/*e* 678.3506, M + H^+^ calcd. for C_35_H_56_NO_8_SSi 678.3496.

**Epoxy Alkene 32.** Epoxy amide **31** (1.20 g, 1.77 mmol, 1.0 equiv) was treated with Red-Al (0.26 mL, 70% *w*/*v* in toluene, 0.89 mmol, 0.5 equiv) and the resulting aldehyde was treated with freshly prepared methylentriphenilfosforane (3.54 mmol, 2.0 equiv) in exactly the same manner as previously described for synthesis of **19** to afford epoxy alkene **32** (640 mg, 72% over two steps) as a white foam: R*_f_* = 0.52 (silica gel, 20% EtOAc in hexanes); [α]^20^_D_ = −27.2 (*c* 1.2, CH_2_Cl_2_); ^1^H NMR (400 MHz, CDCl_3_) *δ* = 0.07 (s, 3 H), 0.09 (s, 3 H), 0.87 (s, 9 H), 1.48 (q, *J* 11.4 Hz, 1 H), 1.53 (s, 3 H), 1.76 (ddd, *J* = 14.4, 7.8, 5.6 Hz, 1 H), 1.81 (q, *J* = 11.6 Hz, 1 H), 2.00 (ddd, *J* = 14.4, 5.0, 3.5 Hz, 1 H), 2.20 (q, *J* = 4.2 Hz, 1 H), 2.24 (q, *J* = 4.5 Hz, 1 H), 3.07 (dd, *J* = 5.4, 2.2 Hz, 1 H), 3.11 (d, *J* = 2.0 Hz, 1 H), 3.15 (ddd, *J* = 11.3, 9.2, 4.5 Hz, 1 H), 3.27 (ddd, *J* = 9.0, 7.8, 3.1 Hz, 1 H), 3.46 (ddd, *J* = 11.7, 9.5, 4.0 Hz, 1 H), 3.55 (ddd, *J* = 10.6, 9.0, 4.7 Hz, 1 H), 3.63 (d, *J* = 3.8 Hz, 1 H), 3.66 (dd, *J* = 2.1, 1.7 Hz, 1 H), 3.96 (d, *J* = 10.0 Hz, 1 H), 5.27 (dd, *J* = 10.1, 1.8 Hz, 1 H), 5.47 (dd, *J* = 17.3, 1.6 Hz, 1 H), 5.57 (s, 1 H), 5.59 (ddd, *J* = 17.3, 10.3, 7.4 Hz, 1 H), 7.38–7.35 (m, 3 H), 7.50–7.48 (m, 2 H); ^13^C NMR (100 MHz, CDC1_3_) *δ* = −4.7, −4.1, 15.1, 17.9, 25.7, 30.5, 33.9, 39.6, 57.6, 58.1, 69.2, 69.9, 70.1, 76.3, 77.8, 79.8, 80.6, 103.0, 119.1, 126.3, 128.4, 129.2, 135.8, 137.5; HRMS (ESI-TOF) *m*/*e* 503.2804, M + H^+^ calcd. for C_28_H_43_O_6_Si 503.2829.

***Tris*-Tetrahydropyran 7.** Epoxy alkene **32** (670 mg, 1.37 mmol) was treated sequentially with TBAF, CSA, and TBSCl in exactly the same manner as previously described for synthesis of **8** to afford *tris*-tetrahydropyran **7** (462 mg, 69% over three steps) as a white solid: R*_f_* = 0.62 (silica gel, 20% EtOAc in hexanes); [α]^20^_D_ = −15.8 (*c* 0.8, CH_2_Cl_2_); ^1^H NMR (400 MHz, CDCl_3_) *δ* = 0.03 (s, 3 H), 0.06 (s, 3 H), 0.87 (s, 9 H), 1.54 (s, 3 H), 1.55 (q, *J* = 11.1 Hz, 1 H), 1.57 (q, *J* = 11.2 Hz, 1 H), 1.83 (q, *J* = 11.6 Hz, 1 H), 2.23 (dt, *J* = 11.3, 4.2 Hz, 1 H), 2.29 (dt, *J* = 11.3, 4.0 Hz, 1 H), 2.35 (dt, *J* = 11.8, 4.2 Hz, 1 H), 3.10 (ddd, *J* = 11.1, 9.0, 3.9 Hz, 1 H), 3.20–3.13 (m, 2 H), 3.47 (ddd, *J* = 10.8, 9.1, 6.2 Hz, 1 H), 3.52 (ddd, *J* = 11.5, 9.4, 4.0 Hz, 1 H), 3.59 (dd, *J* = 8.9, 6.2 Hz, 1 H), 3.69–3.65 (m, 2 H), 3.96 (d, *J* = 10.1 Hz, 1 H), 5.25 (dd, *J* = 10.6, 1.0 Hz, 1 H), 5.36 (dt, *J* = 17.3, 1.4 Hz, 1 H), 5.58 (s, 1 H), 5.85 (ddd, *J* = 17.0, 10.6, 6.2 Hz, 1 H), 7.38–7.35 (m, 3 H), 7.50–7.48 (m, 2 H); ^13^C NMR (100 MHz, CDC1_3_) *δ* = −4.6, −4.2, 15.1, 17.9, 25.7, 30.1, 35.5, 39.3, 69.8, 70.0, 70.5, 76.2, 76.5, 77.2, 78.3, 79.8, 83.3, 103.1, 117.9, 126.3, 128.4, 129.1, 135.8; HRMS (ESI-TOF) *m*/*e* 503.2842, M + H^+^ calcd. for C_28_H_43_O_6_Si 503.2829.

**Epoxy Amides 38a/b.** Aldehyde **23** (750 mg, 1.84 mmol, 1.0 equiv) was dissolved in CH_2_Cl_2_ (30 mL) and *N*,*N*-dimethyl-2-(dimethylsulfuranylidene)acetamide **37** (542 mg, 3.68 mmol, 2.0 equiv) was added at 25 °C. The reaction mixture was stirred at this temperature until the reaction was complete as judged by TLC (ca. 12 h). The solvent was removed by concentration under reduced pressure and the resulting crude product was purified by flash column chromatography (silica gel, 30% EtOAc in hexanes) to obtain epoxy amides **38a/b** as an inseparable 1:1 mixture of diastereoisomers (780 mg, 86% combined yield and a 3:2 dr) as a colorless oil: R*_f_* = 0.42 (silica gel, 50% EtOAc in hexanes); ^1^H NMR (400 MHz, CDCl_3_) (major stereoisomer) *δ* = 0.02 (s, 3 H), 0.03 (s, 3 H), 0.89 (s, 9 H), 1.19 (dd, *J* = 9.0, 8.8 Hz, 1 H), 1.55 (s, 3 H), 1.75–1.64 (m, 1 H), 2.04–1.98 (m, 1 H), 2.74 (ddd, *J* = 14.6, 3.8, 3.6 Hz, 1 H), 2.98 (s, 3 H), 3.12 (s, 3 H), 3.38 (d, *J* = 3.8 Hz, 1 H), 3.65–3.48 (m, 3 H), 3.79 (ddd, *J* = 12.2, 9.1, 4.0 Hz, 1 H), 3.95 (d, *J* = 9.1 Hz, 1 H), 5.75 (s, 1 H), 7.32–7.28 (m, 3 H), 7.43–7.41 (m, 2 H); ^13^C NMR (100 MHz, CDCl_3_) (major stereoisomer) *δ* = −4.9, −4.2, 17.9, 25.6, 28.9, 30.7, 34.4, 38.5, 38.7, 55.4, 57.8, 69.5, 69.8, 76.5, 80.1, 84.3, 95.8, 101.6, 125.6, 126.0, 128.2, 128.9, 137.2, 165.6; HRMS (ESI-TOF) *m*/*e* 492.2775, M + H^+^ calcd. for C_26_H_41_NO_6_Si 492.2781.

**Epoxy Alcohols 39a and 39b.** A solution of epoxy amides **38a/b** (325 mg, 0.66 mmol, 1.0 equiv) in THF (10 mL) was treated with lithium triethylborohydride (Super-H) (1.0 M in THF, 2.00 mL, 2.00 mmol, 3.0 equiv) at 0 °C. The reaction mixture was allowed to warm to room temperature and stirred at this temperature until the reaction was complete, as judged by TLC (ca. 8 h). The excess of Super-H was carefully quenched by the addition of MeOH, and the resulting solution was diluted with Et_2_O (15 mL) and washed with a saturated aqueous NH_4_Cl solution (20 mL). The organic phase was separated, the aqueous layer was extracted with Et_2_O (2 × 10 mL), and the combined organic extracts were washed with brine, dried (MgSO_4_), filtered, and concentrated under reduced pressure. The resulting crude product was purified by flash column chromatography (silica gel, 20% EtOAc in hexanes) to obtain epoxy alcohols **39a/39b** as an inseparable 3:2 mixture of diastereoisomers (275 mg, 92% combined yield and a 1:1 dr) as a colorless oil: R*_f_* = 0.39 (silica gel, 20% EtOAc in hexanes); ^1^H NMR (400 MHz, CDCl_3_) (major stereoisomer) *δ* = 0.03 (s, 3 H), 0.05 (s, 3 H), 0.84 (s, 9 H), 1.19 (dd, *J* = 9.0, 8.8 Hz, 1 H), 1.26 (s, 3 H), 1.73–1.62 (m, 2 H), 2.01–1.95 (m, 1 H), 2.13 (ddd, *J* = 14.6, 3.8, 3.6 Hz, 1 H), 2.37–2.32 (m, 1 H), 2.44–2.39 (m, 1 H), 2.54 (ddd, *J* = 12.8, 7.4, 5.2 Hz, 1 H), 3.55–3.47 (m, 4 H), 3.75 (d, *J* = 9.1 Hz, 1 H), 3.84 (dd, *J* = 9.0, 5.1 Hz, 1 H), 4.00 (d, *J* = 9.1 Hz, 1 H), 5.56 (s, 1 H), 7.32–7.28 (m, 3 H), 7.43–7.41 (m, 2 H); ^13^C NMR (100 MHz, CDCl_3_) (major stereoisomer) *δ* = −4.9, −4.2, 18.9, 25.7, 28.9, 30.7, 34.8, 53.4, 55.8, 65.7, 69.5, 77.5, 78.4, 85.1, 95.8, 101.5, 125.6, 126.0, 128.2, 128.9, 137.2; HRMS (ESI-TOF) *m*/*e* 451.2485, M + H^+^ calcd. for C_24_H_38_O_6_Si 451.2516.

**Epoxy Amide 40.** A solution of aldehyde **30** (150 mg, 0.32 mmol, 1.0 equiv) in CH_2_Cl_2_ (10 mL) was treated with *N*,*N*-dimethyl-2-(dimethylsulfuranylidene)acetamide **37** (145 mg, 0.97 mmol, 3.0 equiv) in exactly the same way as described above for **38**, to obtain, after purification by flash column chromatography (silica gel, 30% EtOAc in hexanes), epoxy amide **40** (144 mg, 82%) as a colorless oil: R*_f_* = 0.47 (silica gel, 50% EtOAc in hexanes); [α]^20^_D_ = –10.5 (*c* 1.5, CH_2_Cl_2_); ^1^H NMR (400 MHz, CDCl_3_) *δ* = 0.02 (s, 3 H), 0.03 (s, 3 H), 0.85 (s, 9 H), 1.19 (dd, *J* = 9.0, 8.7 Hz, 1 H), 1.57 (s, 3 H), 1.73–1.65 (m, 2 H), 2.04–1.99 (m, 2 H), 2.13 (ddd, *J* = 14.6, 3.8, 3.7 Hz, 1 H), 2.97 (s, 3 H), 3.15 (s, 3 H), 3.35–3.31 (m, 1 H), 3.37 (d, *J* = 3.8 Hz, 1 H), 3.85–3.48 (m, 5 H), 4.11 (d, *J* = 9.1 Hz, 1 H), 5.98 (s, 1 H), 7.32–7.28 (m, 3 H), 7.43–7.41 (m, 2 H); ^13^C NMR (100 MHz, CDCl_3_) *δ* = −4.9, −4.2, 19.1, 25.6, 27.5, 30.7, 33.4, 37.5, 38.7, 39.2, 55.7, 57.6, 69.5, 69.7, 77.5, 83.4, 84.6, 89.7, 95.6, 101.2, 125.6, 126.0, 128.2, 128.7, 137.5, 165.5; HRMS (ESI-TOF) *m*/*e* 548.3068, M + H^+^ calcd. for C_29_H_45_NO_7_Si 548.3044.

**Epoxy Alcohol 41.** Epoxy amide **40** (65 mg, 0.12 mmol, 1.0 equiv) in THF (10 mL) was transformed into epoxy alcohol **41** (58 mg, 95%) according to the procedure described before for **39**. Compound **41**: colorless oil; R*_f_* = 0.42 (silica gel, 20% EtOAc in hexanes); [α]^20^_D_ = –10.5 (*c* 1.5, CH_2_Cl_2_); ^1^H NMR (400 MHz, CDCl_3_) *δ* = 0.02 (s, 3 H), 0.03 (s, 3 H), 0.80 (s, 9 H), 1.19 (dd, *J* = 9.0, 8.8 Hz, 1 H), 1.27 (s, 3 H), 1.74–1.65 (m, 2 H), 2.08–1.98 (m, 2 H), 2.13 (ddd, *J* = 14.6, 3.8, 3.6 Hz, 1 H), 2.44–2.32 (m, 1 H), 3.37 (ddd, *J* = 12.2, 9.1, 4.0 Hz, 1 H), 3.67–3.47 (m, 4 H), 3.75 (d, *J* = 9.1 Hz, 1 H), 3.82 (dd, *J* = 9.0, 5.1 Hz, 1 H), 4.01 (d, *J* = 9.1 Hz, 1 H), 5.89 (s, 1 H), 7.32–7.28 (m, 3 H), 7.43–7.41 (m, 2 H); ^13^C NMR (100 MHz, CDCl_3_) *δ* = −4.9, −4.2, 19.1, 25.7, 28.9, 30.7, 34.7, 37.8, 53.4, 59.8, 63.7, 68.2, 69.5, 78.5, 79.4, 83.5, 89.7, 95.8, 101.2, 125.6, 126.0, 128.2, 128.9, 137.3; HRMS (ESI-TOF) *m*/*e* 507.2804, M + H^+^ calcd. for C_27_H_42_O_7_Si 507.2778.

**Epoxy Alkene 32.** Epoxy amide **40** (72 mg, 0.13 mmol, 1.0 equiv) was treated with Red-Al (20.0 μL, 70% *w*/*v* in toluene, 0.07 mmol, 0.5 equiv) and the resulting aldehyde was treated with freshly prepared methylentriphenilfosforane (0.26 mmol, 2.0 equiv) in exactly the same manner as previously described for synthesis of **32** from epoxy amide **31**, to afford epoxy alkene **32** (50 mg, 76% over two steps) as a white foam.

## Data Availability

The original data presented in the study are included in the article/Supplementary Material; further inquiries can be directed to the corresponding author.

## Supplementary Materials

The following supporting information can be downloaded at: https://www.mdpi.com/article/10.3390/md23020051/s1.
