# Supplementary material for: Efficient Synthesis of Fused Polycyclic Ether Systems via Sulfonium Ylides: A Synthetic Approach to Yessotoxin and Adriatoxin
Source: Mar Drugs. 2025 Jan 21;23(2):51. doi: 10.3390/md23020051 (PMC11857374; doi:10.3390/md23020051)
Supplement: Supplementary file 1 [file marinedrugs-23-00051-s001.zip › marinedrugs-3398734-supplementary.pdf]

Supporting Information

Efficient Synthesis of Fused Polycyclic Ether Systems via Sulfonium Ylides: A Synthetic Approach to Yessotoxin and Adriatoxin

Federico Moya-Utrera[1], Iván Cheng-Sánchez[1*], Irama Fuentes-Pino[1], Antonio Sánchez-Ruiz[2] and Francisco Sarabia[1*]

[1] Department of Organic Chemistry, Faculty of Sciences, University of Malaga, 29071 Málaga, Spain
[2] Faculty of Pharmacy, University of Castilla-La Mancha, Campus de Albacete, Avda. Dr. José María Sánchez Ibáñez S/N, 02008 Albacete, Spain
* Correspondence: cheng@uma.es (I. C.-S.); frsarabia@uma.es (F. S.).

Index **Pages**

Index	Pages
1. Theoretical Calculations of Aldehydes **23** and **30**	S2-S16
1. Theoretical Calculations of Transitions States	S16-S21
2. ^{1}H and ^{13}C NMR Spectra of Compounds	S22-S34

1. Theoretical Calculations of Aldehydes 23 and 30

Minimum energy conformations were calculated in vacuo using the PM3 method found in Gaussian'09 software, as the calculation method for the relaxed PES scan. Optimization was performed using Polak-Ribiere algorithm until the RMS gradient reached a value below 0.001 kcal/(Å·mol).

The resulting minimum-energy conformations for compounds **23** and **30** are contained in the following .mol files:

1.1. Aldehyde 23

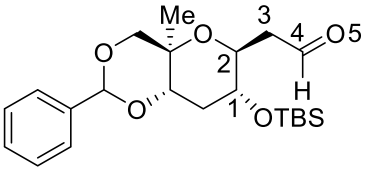

Dihedral 1: 1-2-3-4
Dihedral 2: 2-3-4-5

23

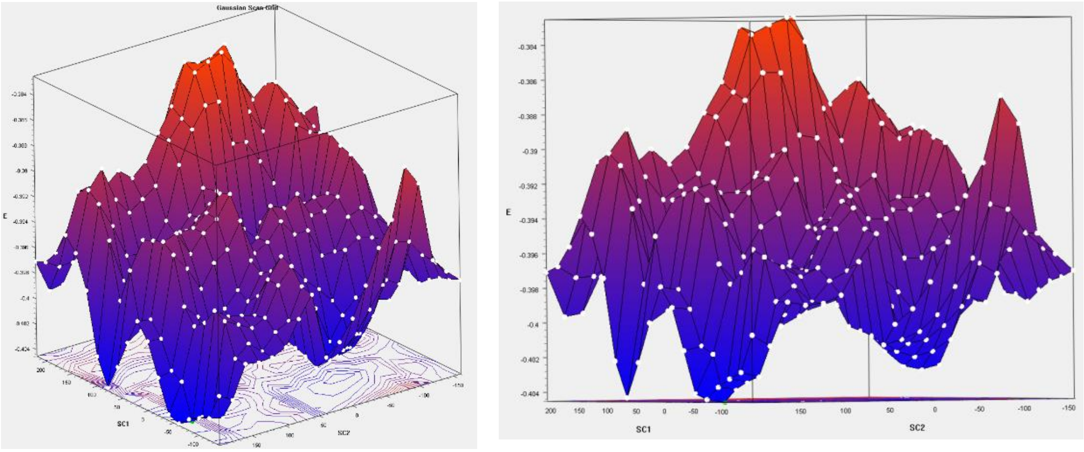

Figure S1. Dihedral 1-Dihedral 2 scan surface for aldehyde **23**

Minimum energy conformer structures:

Conformer 94/361:

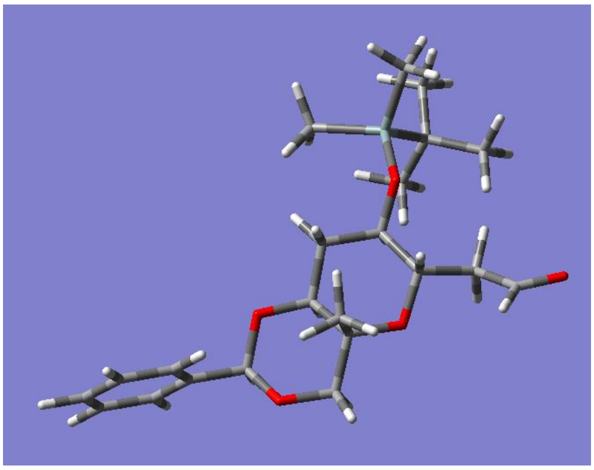

Figure S2. *re* face is more accesible in this conformer (*si* face is more hindered because of the OTBS group).

Conformer 83/361:

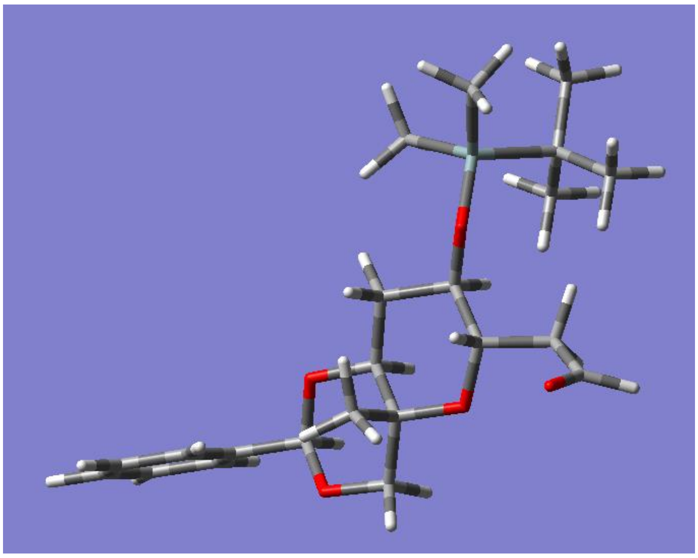

Figure S3. *si* face is more accesible (*re* face suffers the steric hindrance of the TBS moiety).

Conformer 201/361:

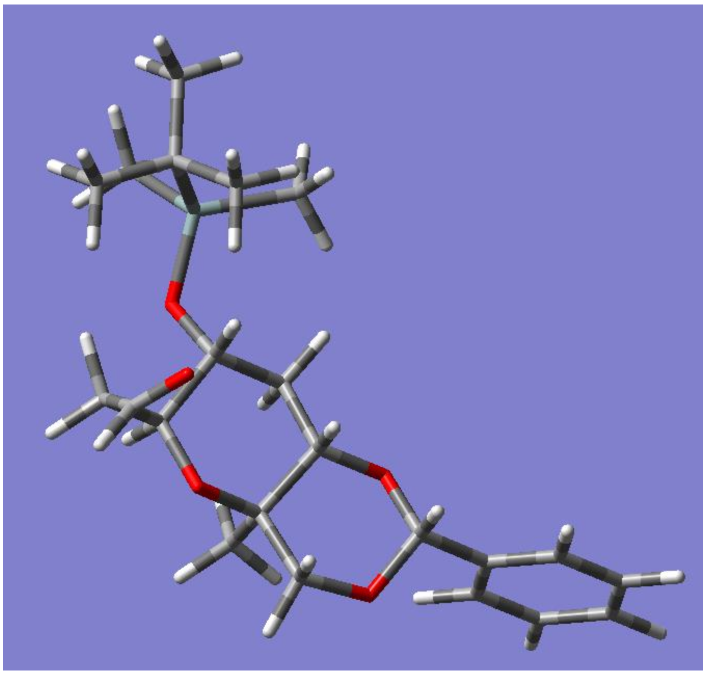

Figure S4. *si* face is more accesible (*re* face suffers the steric hindrance of the pyrane ring).

Conformer 360/361:

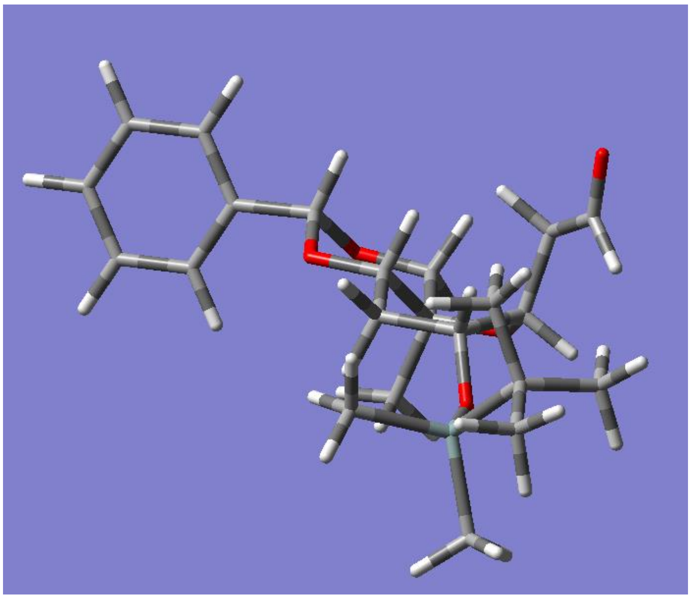

Figure S5. *re* face is more accesible (*si* face is completely blocked by the OTBS group).

Conformer 244/361 (shallow minimum):

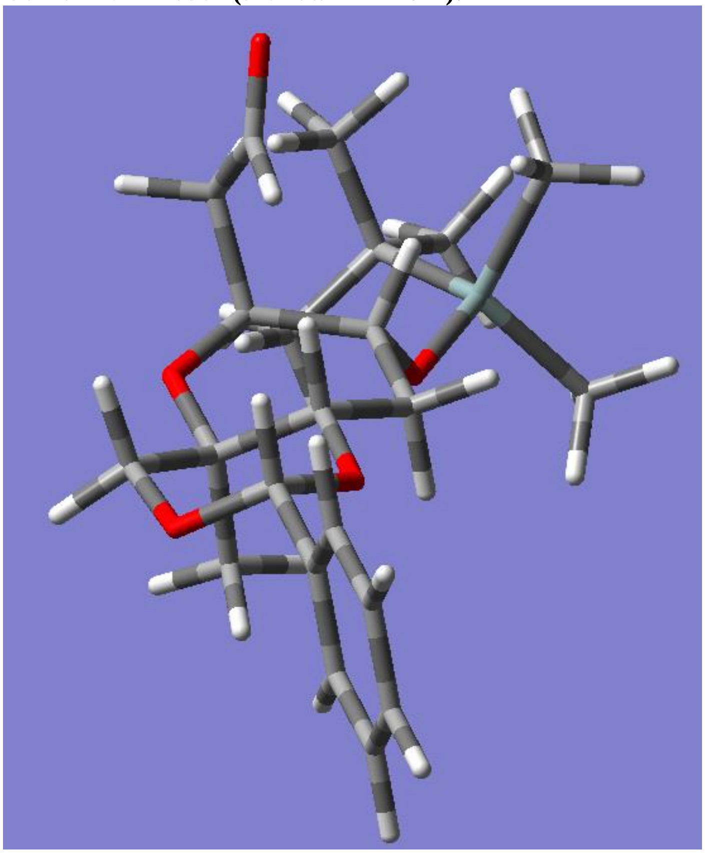

Figure S6. *si* face is more accesible (*re* face is completely blocked by the OTBS group).

Conformer 110/361 (shallow minimum):

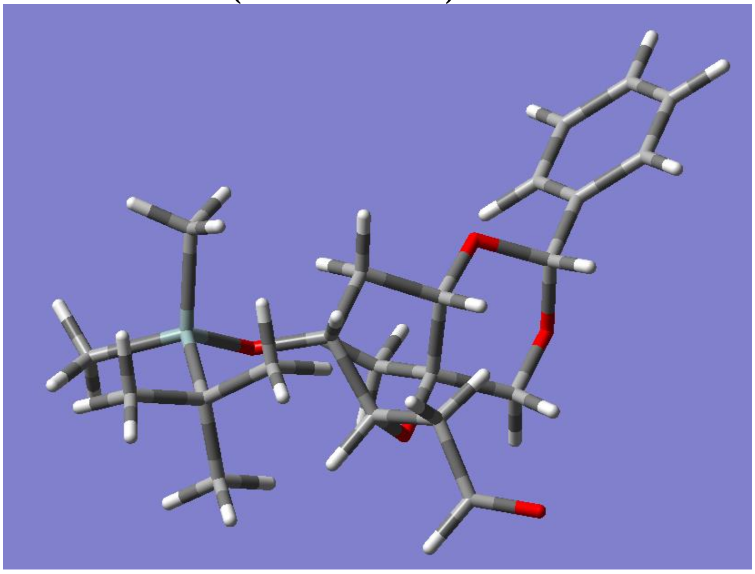

Figure S7. *si* face is slightly more accesible, although both faces are hindered towards the attack of the nucleophile.

Conformer 228/361 (shallow minimum):

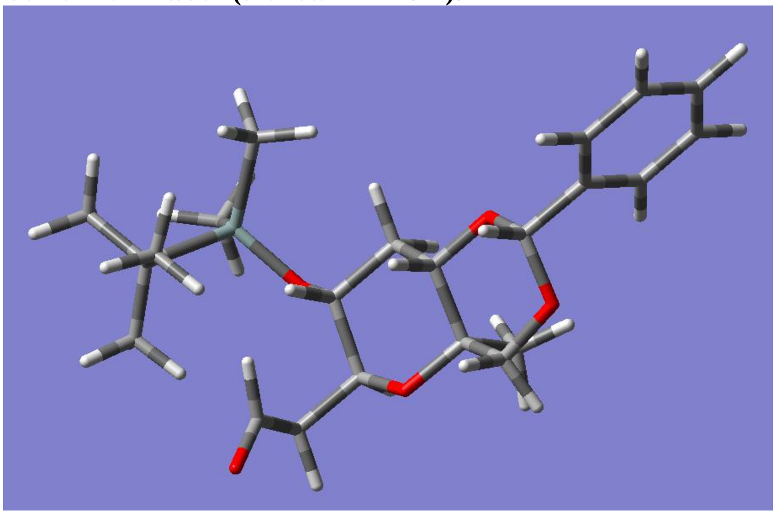

Figure S8. Both faces are blocked towards the attack of the nucleophile.

The inspection of the PES showed that the average value for the maximum potential energy barrier that separate the different minima is of 5.78 Kcal/mol, which allows for the equilibration among the most stable conformers. Inspection of the structures for the different conformers reveal that out of 7 minima (2 of them relatively shallow minima that can quickly evolve towards any of the remaining deeper wells), two of them exposed the *re* face, and one had both faces blocked towards the attack of the nucleophile; the remaining minima exposed the si faces towards the attack. Overall, 4 out of 6 viable conformers exhibited a *si* face preference, which would account for a 66% probability of yielding the **38a** isomer, which fits with the observed experimental stereoselectivity.

Atom coordinates:

Conformer 94/361:

H 2.59411200 3.27832300 -0.51557400
C 2.29489700 2.25625900 -0.79958300
C 3.37203600 0.11670500 -0.80003900
C 1.08580100 0.17513600 -0.23638300
C 1.14783800 1.69807200 0.07938600
H 3.13994300 -0.08202700 -1.87831000
H 2.02931100 2.25064000 -1.87430300
C 1.42665500 1.99567900 1.55650800
H 2.48454600 1.82357700 1.79968900
H 0.83482000 1.33709300 2.21489800
H 1.18923400 3.03706900 1.80783100
O -0.07407900 2.29208900 -0.36605000
H 0.85016800 0.02383000 -1.32253300
C -0.01545400 -0.45260000 0.60419500
H -0.06026900 -1.55605000 0.47757900
H 0.17616200 -0.27501300 1.68699900
C -1.24446700 1.76639700 0.27542500
C -1.34743800 0.20407400 0.17679800
H -1.59617800 -0.08598700 -0.88296400
O -2.37530700 -0.19100700 1.04324600
C -2.41847500 2.44494700 -0.43880300
H -3.33126100 1.81368100 -0.33190200
H -2.23605600 2.51021600 -1.53197200

C -2.67587100 3.83425000 0.09090900
H -1.85366800 4.33387900 0.62936700
O -3.73341900 4.40268500 -0.06203200
H -1.21957600 2.06681000 1.35274100
Si -3.24138600 -1.63087100 0.71445500
C -2.10712700 -3.14691500 0.53894100
H -2.08816300 -3.46536000 -0.51371500
H -2.46370500 -3.98239500 1.14537200
H -1.07745900 -2.91785300 0.84074600
C -4.41810700 -1.81925400 2.18594600
H -3.99928600 -2.46848000 2.95821400
H -5.35999700 -2.26333700 1.83103800
H -4.64552700 -0.85100300 2.63947700
C -4.30117900 -1.53661400 -0.91539900
C -5.24955800 -2.71326800 -0.84658800
H -4.71204300 -3.66894600 -0.92259400
H -5.99364500 -2.68751900 -1.65441500
H -5.79453700 -2.71576900 0.11512600
C -3.34100100 -1.65736300 -2.07438800
H -2.73368400 -2.57571900 -1.97851100
H -2.64048500 -0.80425800 -2.09963700
H -3.86061700 -1.69409200 -3.04083000
C -5.06084600 -0.23341500 -0.93724800
H -5.71266500 -0.13908600 -0.05752300
H -5.69684900 -0.15000700 -1.83029700
H -4.38033300 0.63419500 -0.93888800
C 4.67929000 -0.51384400 -0.37543800
C 7.08985100 -1.69182800 0.36962600
C 5.40306400 -1.26827300 -1.29917400
C 5.16472200 -0.34985900 0.92192900
C 6.36929400 -0.93963300 1.29094300
C 6.60625100 -1.85542800 -0.92454600
H 5.02650200 -1.39954900 -2.32016700
H 4.59376100 0.24535200 1.64517500
H 6.74969700 -0.81004700 2.30936600
H 7.17362800 -2.44709500 -1.65022500
H 8.03779300 -2.15505800 0.66202600
O 2.34798300 -0.42565800 0.03427300
O 3.48415500 1.52099600 -0.57840600

Conformer 83/361:
H 2.61108400 3.18891900 -0.74442200
C 2.29543200 2.15579700 -0.96330300
C 3.33780700 0.00317300 -0.81864700
C 1.05101000 0.13558000 -0.27084500
C 1.13718600 1.67500700 -0.05436000
H 3.10770900 -0.26386700 -1.88250500
H 2.03312300 2.08532200 -2.03649900
C 1.41719200 2.06385800 1.40105300
H 2.47295000 1.89819800 1.65716800
H 0.81798600 1.45432800 2.09889400
H 1.18766700 3.12117900 1.58474700
O -0.07461200 2.25460000 -0.54443800
H 0.81741100 -0.08253700 -1.34604300
C -0.06458300 -0.41844500 0.60258200
H -0.12804200 -1.52659400 0.54568100
H 0.12577300 -0.17432600 1.67257200
C -1.24967600 1.79830500 0.14138600
C -1.38210000 0.23440400 0.12715700
H -1.62642600 -0.10969800 -0.91738100
O -2.42559100 -0.09681900 1.00205100
C -2.41252700 2.45630200 -0.60612400

H -3.34653100 1.87524400 -0.42072200
H -2.24807700 2.40820100 -1.70277500
C -2.69640800 3.88551600 -0.22299800
H -3.29254300 4.46163600 -0.95116300
O -2.34426700 4.41379000 0.80563900
H -1.21109500 2.15750500 1.20001600
Si -3.31132800 -1.53767700 0.74064700
C -2.20691300 -3.08037900 0.62043500
H -2.19042500 -3.43509200 -0.42054200
H -2.58194800 -3.88753400 1.25355700
H -1.17423300 -2.86074100 0.91908500
C -4.48695600 -1.64753300 2.22106300
H -4.07840200 -2.27730600 3.01472500
H -5.43947600 -2.08416900 1.88607000
H -4.69132300 -0.65901500 2.64055000
C -4.37650700 -1.48717700 -0.88854600
C -5.34800900 -2.64082500 -0.77252000
H -4.82968200 -3.60910800 -0.81414900
H -6.09478300 -2.63088000 -1.57820900
H -5.88869400 -2.59631200 0.19060100
C -3.42267300 -1.67077700 -2.04453600
H -2.83440700 -2.59746900 -1.91563500
H -2.70394600 -0.83448300 -2.10231000
H -3.94541600 -1.73250800 -3.00800700
C -5.10997000 -0.17083400 -0.95837400
H -5.76419400 -0.03456200 -0.08596300
H -5.73932000 -0.10413700 -1.85752900
H -4.41136100 0.68215400 -0.98399800
C 4.63306000 -0.61797400 -0.34542500
C 7.02089700 -1.78063600 0.49127700
C 5.34806800 -1.44572500 -1.21132500
C 5.11585700 -0.37293200 0.94011500
C 6.30908500 -0.95530100 1.35496400
C 6.53991300 -2.02507600 -0.79105300
H 4.97352600 -1.64076200 -2.22279200
H 4.55164700 0.27997500 1.61757700
H 6.68742100 -0.76201400 2.36400900
H 7.10041900 -2.67430900 -1.47137900
H 7.95989900 -2.23782500 0.81969700
O 2.30172100 -0.46810100 0.04347700
O 3.47239800 1.41704400 -0.69220800

Conformer 201/361:

H 2.71371400 3.25796000 0.49562600
C 2.35781000 2.37183600 -0.05470500
C 3.31404800 0.26548100 -0.67080900
C 1.03861400 0.28017000 -0.06217000
C 1.18417300 1.65232700 0.65677300
H 3.06126700 0.38833500 -1.75574800
H 2.08853800 2.67807300 -1.08398800
C 1.47901100 1.52213500 2.15471400
H 2.52201700 1.22051600 2.32466800
H 0.84121800 0.75251700 2.62164100
H 1.31321500 2.47091600 2.68020000
O -0.00112600 2.41208500 0.41241100
H 0.78354300 0.44375800 -1.14212100
C -0.08343100 -0.50045700 0.60322100
H -0.18389300 -1.52457400 0.18291500
H 0.12906200 -0.63361300 1.68863000
C -1.20586000 1.78970800 0.87246300
C -1.38717000 0.30300600 0.40219900
H -1.69110000 0.28431000 -0.68243400

O -2.40463800 -0.23995100 1.20231500
C -2.34195100 2.69071000 0.36584000
H -2.47960600 3.53218000 1.07259300
H -3.29677300 2.11588900 0.39060300
C -2.13731200 3.25857600 -1.02043000
H -2.12816200 4.35756200 -1.10557500
O -2.00148200 2.58427800 -2.01550400
H -1.19269400 1.81539700 1.99082600
Si -3.35395500 -1.52833500 0.59535200
C -2.31879900 -2.98282900 -0.05897800
H -2.38176500 -3.00463200 -1.15695300
H -2.68623200 -3.93635100 0.32662900
H -1.26174100 -2.88415800 0.21737200
C -4.42555700 -2.05962000 2.06475000
H -3.98350900 -2.90000800 2.60469400
H -5.41194800 -2.36826700 1.68778500
H -4.57007600 -1.23776600 2.77059000
C -4.52766400 -0.97937300 -0.85493600
C -5.52861800 -2.10153100 -1.01717600
H -5.04950500 -3.01753900 -1.39041000
H -6.32975000 -1.83759200 -1.72098200
H -5.99771500 -2.34560500 -0.04642600
C -3.65615900 -0.81717100 -2.07730200
H -3.10260500 -1.75123800 -2.28328800
H -2.90627700 -0.01985800 -1.92759800
H -4.24067900 -0.56612000 -2.97198500
C -5.21667600 0.30608900 -0.46951200
H -5.81774000 0.17710300 0.44140600
H -5.89045700 0.65826500 -1.26380900
H -4.49053700 1.11289000 -0.27508900
C 4.58820900 -0.52448200 -0.47120500
C 6.93625400 -1.98530200 -0.14429500
C 5.26040300 -1.02531800 -1.58636900
C 5.09388700 -0.75613100 0.80798300
C 6.26709200 -1.48619600 0.96788000
C 6.43244200 -1.75442900 -1.42033800
H 4.86748900 -0.84573200 -2.59380200
H 4.56328900 -0.35973300 1.68241200
H 6.66329600 -1.66719200 1.97229900
H 6.95918800 -2.14694500 -2.29613200
H 7.85949300 -2.55960700 -0.01628800
O 2.26982100 -0.43319100 0.00623500
O 3.50571700 1.54357300 -0.06780500

Conformer 360/361:

H 2.61221000 1.96611000 2.65058100
C 2.35733500 1.75709400 1.59893200
C 3.44093300 0.62757000 -0.21061200
C 1.10068800 0.39688300 -0.03520600
C 1.11181400 0.84568600 1.45064600
H 3.34117800 1.49618700 -0.91182200
H 2.22069600 2.72034400 1.06997900
C 1.20378300 -0.33735000 2.42135200
H 2.20006800 -0.79965900 2.38819400
H 0.46928900 -1.11629400 2.15797000
H 1.01243700 -0.02305500 3.45507800
O -0.06265300 1.59461500 1.78951000
H 0.99437000 1.29557400 -0.70704400
C -0.07942300 -0.53658600 -0.25420100
H -0.11245800 -0.90815900 -1.29557900
H 0.03661900 -1.43874500 0.39226900

C -1.14197400 1.56232100 0.86955700
C -1.38215100 0.21811100 0.10115700
H -1.93458200 0.47130500 -0.84795900
O -2.19016300 -0.57684400 0.92401600
C -1.02336300 2.74604500 -0.11118400
H -0.20844700 2.55677200 -0.84627800
H -0.71242500 3.65837100 0.43904200
C -2.32854300 3.04171300 -0.80444800
H -3.25580000 2.65884100 -0.32858100
O -2.38859600 3.66669600 -1.83900500
H -1.98854700 1.75460200 1.57432400
Si -3.39792100 -1.56385500 0.22253300
C -2.66564600 -2.83100100 -0.98624800
H -2.87157100 -2.51667400 -2.01981500
H -3.10669200 -3.81963800 -0.83674600
H -1.58198100 -2.91997800 -0.86566100
C -4.22034100 -2.41549100 1.70083200
H -3.82595400 -3.42250000 1.85554800
H -5.30197400 -2.49100800 1.51611300
H -4.06881200 -1.84444500 2.62066300
C -4.74299900 -0.53786500 -0.74205800
C -5.92320200 -1.46922300 -0.90736900
H -5.68276500 -2.30577800 -1.57823800
H -6.79810600 -0.95214800 -1.32494300
H -6.21881200 -1.90060300 0.06624600
C -4.14870000 -0.15218800 -2.07588800
H -3.75734900 -1.04262500 -2.60025400
H -3.30306000 0.54629800 -1.94634500
H -4.88402700 0.33164200 -2.73218200
C -5.11680700 0.67022200 0.08254200
H -5.44701600 0.37774200 1.08880000
H -5.93463100 1.24024500 -0.38170800
H -4.26531100 1.36402000 0.20047800
C 4.68967600 -0.18077800 -0.48279900
C 6.99705500 -1.64562400 -1.01477100
C 5.52357800 0.18854700 -1.53846700
C 5.01300100 -1.28461300 0.30635800
C 6.16651000 -2.01423900 0.03791700
C 6.67500000 -0.54466300 -1.80195700
H 5.27437500 1.05550700 -2.16117300
H 4.35539700 -1.57090000 1.13655600
H 6.41980900 -2.88015400 0.65814500
H 7.32894600 -0.25401200 -2.63048500
H 7.90456400 -2.22112500 -1.22421000
O 2.32655900 -0.25338400 -0.35474400
O 3.51784100 1.09397700 1.13385600

Conformer 244/361:

H 2.34645500 1.59727000 2.80280800
C 2.23163400 1.49618400 1.71123500
C 3.55198900 0.57930800 -0.05278100
C 1.21443200 0.27004600 -0.18833200
C 1.02646000 0.60093400 1.31522100
H 3.49764100 1.51852400 -0.66209700
H 2.15177600 2.50814200 1.26878500
C 0.98685300 -0.64509900 2.20586400
H 1.98173500 -1.10582300 2.27820700
H 0.30268900 -1.40590700 1.79571500
H 0.65464300 -0.39871200 3.22208500
O -0.15285300 1.37874400 1.50870500
H 1.13692800 1.21494500 -0.79775500
C 0.12595600 -0.70106700 -0.61161700

H 0.23608200 -0.99139200 -1.67319500
H 0.22172600 -1.64539500 -0.02476300
C -1.29994900 1.07653300 0.71751300
C -1.25518600 -0.05513700 -0.37038300
H -1.61658200 0.38155100 -1.34498500
O -2.10936300 -1.08783200 0.03317000
C -1.77754000 2.41099000 0.11329700
H -1.73427600 3.20999100 0.88297900
H -2.85744600 2.31285700 -0.14658800
C -0.98365100 2.88165100 -1.07726200
H 0.03931400 2.47283800 -1.19874300
O -1.41165600 3.68768600 -1.87240500
H -2.04788000 0.78157000 1.50795000
Si -3.66625200 -1.26072600 -0.65763900
C -3.73913700 -0.52883400 -2.40922100
H -4.57429800 0.18082600 -2.48888100
H -3.86870000 -1.30405300 -3.16724500
H -2.81092200 0.01741100 -2.62717200
C -3.92366200 -3.14001200 -0.68796100
H -3.95016400 -3.51824500 -1.71312800
H -4.87199200 -3.40344600 -0.19960500
H -3.11520300 -3.65103000 -0.15753900
C -5.02908100 -0.41538000 0.45765500
C -6.29050000 -1.23534200 0.30291200
H -6.70548900 -1.14409900 -0.71038100
H -7.07103700 -0.91675500 1.00804300
H -6.09534900 -2.30710000 0.48272500
C -5.24822000 0.99509900 -0.03746100
H -5.40332300 1.01048500 -1.13102100
H -4.38155400 1.64217500 0.17739800
H -6.12646000 1.45987400 0.43153500
C -4.53303900 -0.43308400 1.88423600
H -4.36611100 -1.46004500 2.23634000
H -5.25307500 0.03741800 2.56900400
H -3.57726900 0.11212400 1.98663500
C 4.85480500 -0.15850600 -0.26407500
C 7.26547700 -1.48811500 -0.68118500
C 5.78839700 0.35396500 -1.16531000
C 5.13026200 -1.33770400 0.42821500
C 6.33559700 -1.99944100 0.21748400
C 6.99117800 -0.31190300 -1.37173100
H 5.57720000 1.28054700 -1.71151300
H 4.39454400 -1.73723400 1.13691000
H 6.55107500 -2.92479000 0.76152800
H 7.72323500 0.09108000 -2.07904600
H 8.21353300 -2.01047900 -0.84545300
O 2.49623800 -0.30858400 -0.41382400
O 3.45108800 0.88680300 1.33429700

Conformer 110/361:

H 2.36114400 2.20877100 2.22398700
C 2.16808400 1.79592700 1.22055500
C 3.40796000 0.45441300 -0.32307300
C 1.08567500 0.07189700 -0.17776700
C 1.00035300 0.77602700 1.20318600
H 3.27508300 1.17650900 -1.16988300
H 1.98614400 2.63515000 0.52138600
C 1.13667600 -0.20382200 2.37430900
H 2.16242400 -0.59087500 2.44822900
H 0.46618100 -1.06881200 2.24060600
H 0.88844200 0.27639900 3.32918500
O -0.24169100 1.47360300 1.36899400

H 0.94120800 0.82962300 -1.00025700
C -0.01461300 -0.97551800 -0.25773900
H 0.03662000 -1.55140900 -1.20042300
H 0.11658500 -1.71779800 0.56453300
C -1.26856400 1.20509400 0.43210700
C -1.37514400 -0.25606100 -0.13057100
H -1.84601700 -0.16961700 -1.15149600
O -2.19970500 -1.00309300 0.72156800
C -1.21669300 2.19654400 -0.74662000
H -2.04094300 1.94473000 -1.45588900
H -0.26748200 2.06947800 -1.31342500
C -1.41567700 3.62162600 -0.29643600
H -2.18251000 3.79635100 0.47669900
O -0.78652400 4.54638200 -0.75613300
H -2.17199600 1.43201600 1.06163000
Si -3.67175000 -1.60417700 0.08695200
C -3.33722000 -2.75705800 -1.38419900
H -3.61323600 -2.25017100 -2.32039100
H -3.92033700 -3.67808900 -1.30989600
H -2.27922900 -3.02827100 -1.44318500
C -4.50714300 -2.51147900 1.52031000
H -4.31578900 -3.58653700 1.48416100
H -5.59371300 -2.35008400 1.46060600
H -4.15725100 -2.13484500 2.48516100
C -4.84978000 -0.18312100 -0.53783100
C -6.21150700 -0.82192400 -0.69617200
H -6.22121500 -1.54932200 -1.51982900
H -6.99180100 -0.07699600 -0.90395300
H -6.49680100 -1.36201200 0.22512600
C -4.30631100 0.30425100 -1.86022000
H -4.18985700 -0.53868400 -2.56534900
H -3.30404800 0.76353200 -1.73990600
H -4.96188400 1.05039800 -2.32776200
C -4.88157700 0.91009300 0.50291300
H -5.27360400 0.53565800 1.45873000
H -5.51973300 1.74895700 0.19017800
H -3.87387800 1.31829000 0.69836500
C 4.72291700 -0.28733500 -0.41077000
C 7.15234400 -1.63371600 -0.60597900
C 5.57645600 -0.03588200 -1.48522600
C 5.08802400 -1.21353000 0.56627800
C 6.30237600 -1.88452200 0.46568600
C 6.78884400 -0.70959500 -1.58031000
H 5.29499300 0.69227500 -2.25468700
H 4.41493400 -1.40685600 1.41079300
H 6.58832300 -2.61092900 1.23327400
H 7.45828100 -0.51119200 -2.42353900
H 8.10781500 -2.16261500 -0.68304500
O 2.36781500 -0.52435800 -0.34163100
O 3.39195100 1.15141700 0.91949600

Conformer 228/361:

H 2.66887000 3.26429600 0.49805600
C 2.33378900 2.37076200 -0.05347700
C 3.34545800 0.29519000 -0.68737000
C 1.07509900 0.24190100 -0.06036400
C 1.18824200 1.61446200 0.66493800
H 3.08240700 0.41541100 -1.77014700
H 2.04688900 2.67435300 -1.07874700
C 1.50252700 1.48261500 2.15864500
H 2.55829300 1.22123700 2.31641700
H 0.90077200 0.68378200 2.62450400

H 1.30452900 2.41956400 2.69425500
O -0.02315000 2.33682800 0.43188300
H 0.80726100 0.40488500 -1.13733700
C -0.02089100 -0.57366300 0.60818900
H -0.10612300 -1.59301400 0.17295200
H 0.20971300 -0.71695200 1.68847900
C -1.19606800 1.68611200 0.93572100
C -1.34369200 0.20411800 0.43964600
H -1.64995100 0.22784500 -0.65004200
O -2.34511900 -0.39064400 1.21924300
C -2.36646400 2.55410800 0.45643900
H -2.33102300 3.54606500 0.94837400
H -3.32276900 2.07993600 0.77863300
C -2.40974300 2.68164400 -1.04634300
H -2.21392600 1.73743100 -1.60599000
O -2.68003000 3.70810700 -1.62426500
H -1.15452100 1.70458600 2.05246700
Si -3.30086500 -1.63146300 0.52668400
C -2.26662100 -2.98957700 -0.30903300
H -2.34334800 -2.88323100 -1.40109200
H -2.62365400 -3.98481900 -0.03476900
H -1.20673900 -2.91742300 -0.03448600
C -4.31902300 -2.32362700 1.96400100
H -3.85033000 -3.20703500 2.40363600
H -5.31285200 -2.60804800 1.58722400
H -4.45202300 -1.57897600 2.75308700
C -4.52339300 -0.94516700 -0.82165300
C -5.51645600 -2.05820000 -1.07332100
H -5.04014200 -2.92020100 -1.56111700
H -6.34476800 -1.73039000 -1.71614700
H -5.94837600 -2.41439300 -0.12007600
C -3.70081800 -0.64034700 -2.05080000
H -3.12334500 -1.53072500 -2.35898100
H -2.96929700 0.17038000 -1.85507600
H -4.32365400 -0.32909000 -2.89957200
C -5.21411000 0.27945400 -0.27363600
H -5.80914900 0.03330100 0.61692400
H -5.89443300 0.72624100 -1.01271200
H -4.49012100 1.05905400 0.02081000
C 4.64208600 -0.45985500 -0.49897000
C 7.03193100 -1.85564800 -0.19239000
C 5.32049800 -0.93817500 -1.62025400
C 5.16256600 -0.68110700 0.77611700
C 6.35665900 -1.37877000 0.92582700
C 6.51342500 -1.63487300 -1.46436400
H 4.91651500 -0.76593400 -2.62455000
H 4.62744400 -0.30182400 1.65539000
H 6.76465300 -1.55146900 1.92701800
H 7.04528100 -2.00950500 -2.34491600
H 7.97176000 -2.40426400 -0.07239200
O 2.32485300 -0.43807900 -0.00912000
O 3.50447800 1.57519800 -0.08010900

1.2. Aldehyde 30

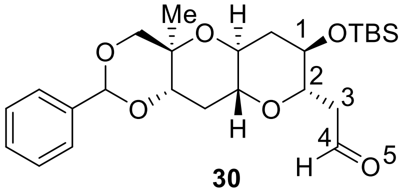

30

Dihedral 1: 1-2-3-4
Dihedral 2: 2-3-4-5

Dihedral 1-Dihedral 2 scan surface.

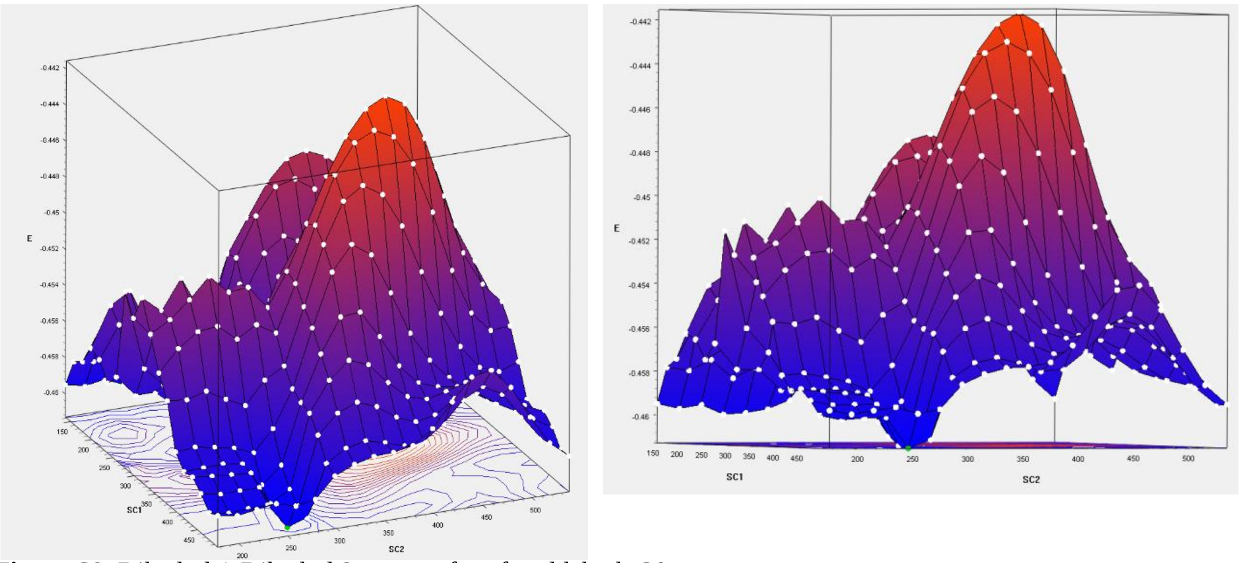

Figure S9. Dihedral 1-Dihedral 2 scan surface for aldehyde **30**

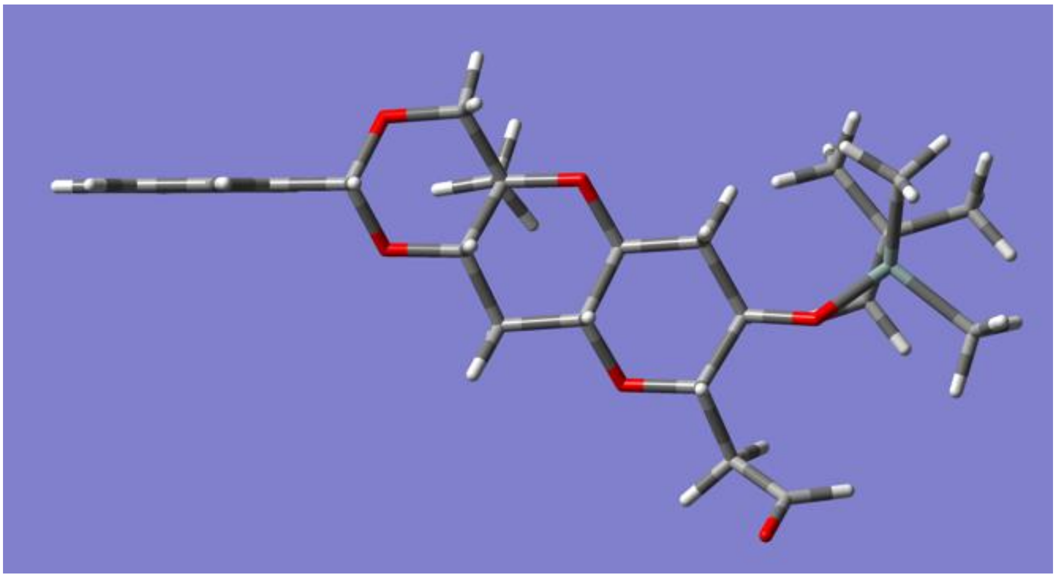

Figure S10. *si* face is more accesible (*re* face suffers the steric hindrance of the TBS moiety).

Atom coordinates:
Minimum energy conformer

H	-2.91043300	-2.86788100	-1.04125400
C	-2.99978600	-1.77732100	-1.17548000
C	-4.73236400	-0.16264300	-0.81449000
C	-2.51294800	0.50946400	-0.40303100
C	-2.03268200	-0.97073000	-0.27270600
H	-4.69809400	0.22376100	-1.86598600

H	-2.85282800	-1.54243200	-2.24751000
C	-2.08520100	-1.51931000	1.15744500
H	-3.05672000	-1.31173800	1.62598900
H	-1.30051500	-1.07506100	1.79192100
H	-1.93345900	-2.60681100	1.16698700
O	-0.70835900	-1.07413300	-0.81927100
H	-2.46153000	0.81260100	-1.48296600
C	-1.61648800	1.45182400	0.39809000
H	-1.94895900	2.50246000	0.29701100
H	-1.65181200	1.22804300	1.48285200
C	0.20767000	-0.23583500	-0.10834200
C	-0.20472000	1.26123900	-0.17562300
H	-0.17848900	1.62014800	-1.23579300
O	0.70322900	2.03345200	0.60083800
C	1.59082000	-0.39652600	-0.73005500
H	1.57253800	-0.16950200	-1.81472000
H	0.22307000	-0.57122900	0.96494900
C	-6.12222300	-0.08008000	-0.22474100
C	-8.69558800	0.08805500	0.82560200
C	-7.16300000	0.42619300	-1.00374500
C	-6.37217600	-0.50213000	1.08103500
C	-7.65878300	-0.41668700	1.60283600
C	-8.44687800	0.50897000	-0.47688600
H	-6.97205200	0.75928600	-2.03044900
H	-5.55150700	-0.89955700	1.69096200
H	-7.85386600	-0.74791900	2.62795900
H	-9.26293600	0.90650100	-1.08896000
H	-9.70752800	0.15452300	1.23818300
O	-3.86743500	0.60714900	0.02219100
O	-4.33964000	-1.53389200	-0.78836900
H	1.94039300	-1.45167800	-0.64369800
C	2.57432400	0.52879300	0.01331600
C	2.03492200	1.99690200	0.07463900
H	2.02139100	2.44166800	-0.95137200
H	2.72072600	0.14244800	1.05985300
O	3.80261600	0.60022100	-0.64732600
Si	4.93841000	-0.67613400	-0.60918300
C	4.74917000	-1.68453000	-2.20302200
H	3.72142700	-2.04255400	-2.32041800
H	5.41081800	-2.55613600	-2.19393600
H	4.99759500	-1.07880800	-3.07878600
C	4.90614700	-1.87102800	0.93013100
C	4.78224200	-1.03599900	2.18329000
H	3.82705900	-0.48497700	2.21164300
H	5.59233700	-0.29629100	2.24990200
H	4.82689100	-1.65672600	3.08980000
C	3.75201100	-2.83318200	0.77825300
H	2.79529000	-2.30111500	0.63496000
H	3.64351900	-3.48035400	1.65970100
H	3.88911500	-3.48540700	-0.09654900
C	6.23430900	-2.59684700	0.90015800
H	7.07258600	-1.87832200	0.85270300
H	6.31606100	-3.25379500	0.02248700
H	6.37802800	-3.22030900	1.79334900
C	2.87190700	2.86211200	1.03008300
H	3.39007600	2.23054400	1.77927900
H	2.21761700	3.54067500	1.61740000
C	3.88922500	3.67565100	0.26082200
H	4.92908700	3.30705700	0.27004700
C	6.60198500	0.24468800	-0.61031800
H	7.00456500	0.34410600	-1.62092500
H	7.32913300	-0.30981800	-0.00041000

H	6.49254700	1.24830800	-0.18833000
O	3.60705700	4.67544000	-0.35817400

This aldehyde shows a PES with one minima, which exposed the *si* face of the aldehyde towards the attack of the nucleophile, yielding only one diastereoisomer, as it is experimentally observed.

2. Theoretical Calculations of Transition States

The energies of the transition states were similarly calculated using the PM3 method found in Gaussian’09 software and the optimization was performed using Polak-Ribiere algorithm until the RMS gradient reached a value below 0.001 kcal/(Å·mol).

2.1. Transition state [A]$^{\neq}$

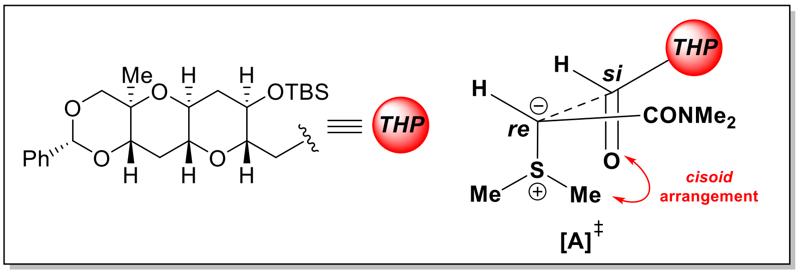

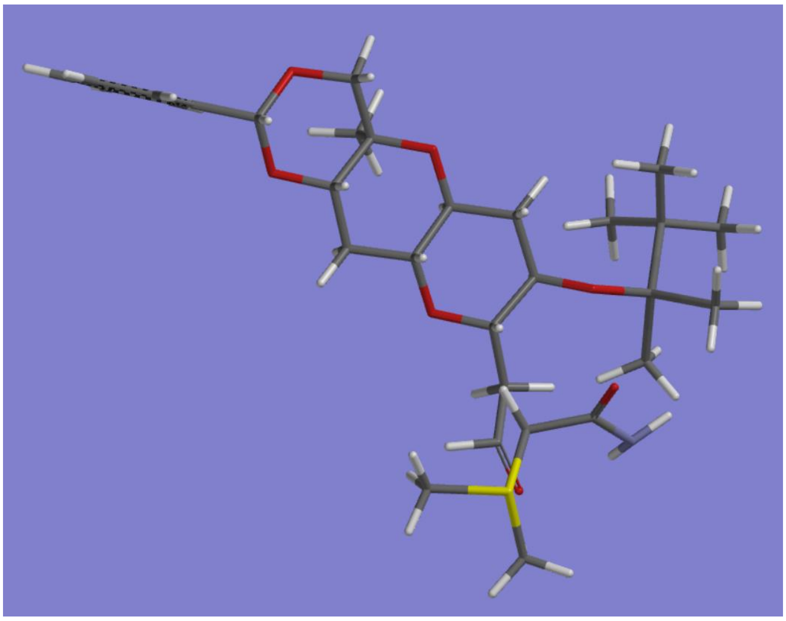

Figure S11. Transition state **[A]**$^{\neq}$ (Approach Ylide *re*-Aldehyde *si*)

Atom coordinates (TS *re-si* **[A]**$^{\neq}$):

H	-4.138341	-1.229923	3.445019
C	-4.128775	-1.345122	2.348554
C	-5.733656	-1.041213	0.598414
C	-3.497143	-0.489043	0.125263
C	-3.144787	-0.378555	1.642810
H	-5.594981	-2.078348	0.195475
H	-3.907068	-2.403361	2.108918
C	-3.304758	1.033394	2.216795
H	-4.271867	1.469568	1.932761
H	-2.508941	1.708238	1.860124
H	-3.251161	1.020130	3.313619
O	-1.811370	-0.872108	1.841004
H	-3.366722	-1.556487	-0.198902
C	-2.564074	0.378193	-0.718386
H	-2.809272	0.302145	-1.794558
H	-2.658457	1.452613	-0.463894
C	-0.860478	-0.105588	1.095322
C	-1.150737	-0.151428	-0.431287
H	-1.064920	-1.204127	-0.803984
O	-0.209157	0.659721	-1.122341
C	0.525982	-0.686693	1.347443
H	0.567557	-1.756088	1.056908
H	-0.912630	0.958251	1.455508
C	-7.150987	-0.549650	0.411336
C	-9.781328	0.301844	0.079416
C	-8.099092	-1.409376	-0.144593
C	-7.521430	0.738077	0.798431
C	-8.836378	1.160457	0.630652
C	-9.411775	-0.982137	-0.308615
H	-7.813023	-2.422591	-0.449779
H	-6.773506	1.411010	1.235864
H	-9.126911	2.170966	0.935946
H	-10.155410	-1.658747	-0.742103
H	-10.816415	0.635051	-0.048487
O	-4.860872	-0.131661	-0.074197
O	-5.461967	-1.021984	1.998509
H	0.781789	-0.663695	2.430455
C	1.552086	0.138978	0.541634
C	1.134426	0.183714	-0.968463
H	1.184225	-0.856088	-1.390256
H	1.594465	1.180620	0.963811
O	2.796323	-0.494400	0.631694
Si	4.108427	0.222087	1.464217
C	5.429847	-1.132091	1.307170
H	5.060210	-2.090462	1.682176
H	6.333359	-0.873381	1.866048
H	5.704004	-1.261170	0.250258
C	3.710545	0.655233	3.314387
C	2.758832	1.830570	3.314118
H	1.892968	1.645757	2.653400
H	3.250345	2.746306	2.955634
H	2.369853	2.039357	4.320486
C	3.088073	-0.562170	3.956988
H	2.111540	-0.803496	3.503302
H	2.918141	-0.409336	5.032559
H	3.732483	-1.445587	3.846165
C	5.019115	1.011396	3.982842
H	5.515055	1.852365	3.477056
H	5.717268	0.162274	3.975716

H	4.868351	1.303512	5.032085
C	2.022395	1.136674	-1.769044
H	2.981722	1.291503	-1.224207
H	1.565301	2.148345	-1.806595
C	4.748452	1.771227	0.567014
H	5.665971	1.542639	0.015416
H	4.971598	2.581308	1.267944
H	4.010996	2.144331	-0.156635
C	2.675977	-0.877410	-3.343514
H	1.781077	-1.418173	-2.939398
S	2.808961	-1.406344	-5.113439
C	1.157583	-0.977668	-5.722375
H	0.468422	-1.804598	-5.510165
H	1.168323	-0.807888	-6.805505
H	0.759201	-0.066777	-5.234830
C	3.883938	-1.376321	-2.568474
N	4.984763	-0.535127	-2.323444
H	5.479927	-0.753052	-1.477952
H	4.800642	0.441533	-2.436832
O	3.954065	-2.538729	-2.175644
C	2.331404	0.735510	-3.235623
H	1.368467	0.784904	-3.834183
O	3.288805	1.468149	-3.758329
C	3.853021	-0.158722	-5.882926
H	4.893979	-0.498028	-5.884433
H	3.803152	0.804043	-5.255301
H	3.544943	0.062953	-6.907976

This transition state, confirmed by the presence of a single imaginary vibration mode at the frequency of 36 cm^{-1}, presented an energy of −1155.82 KJ/mol, and was reached from the minimun energy conformer.

2.2. Transition state [B]$^{\neq}$

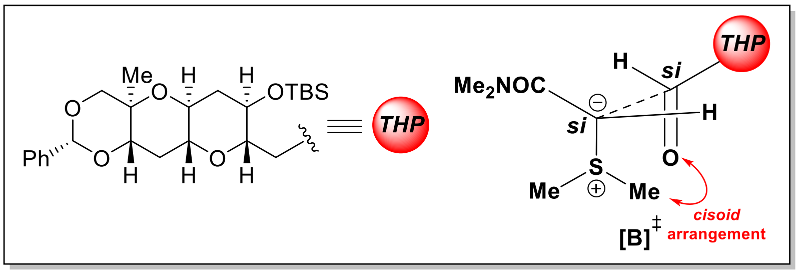

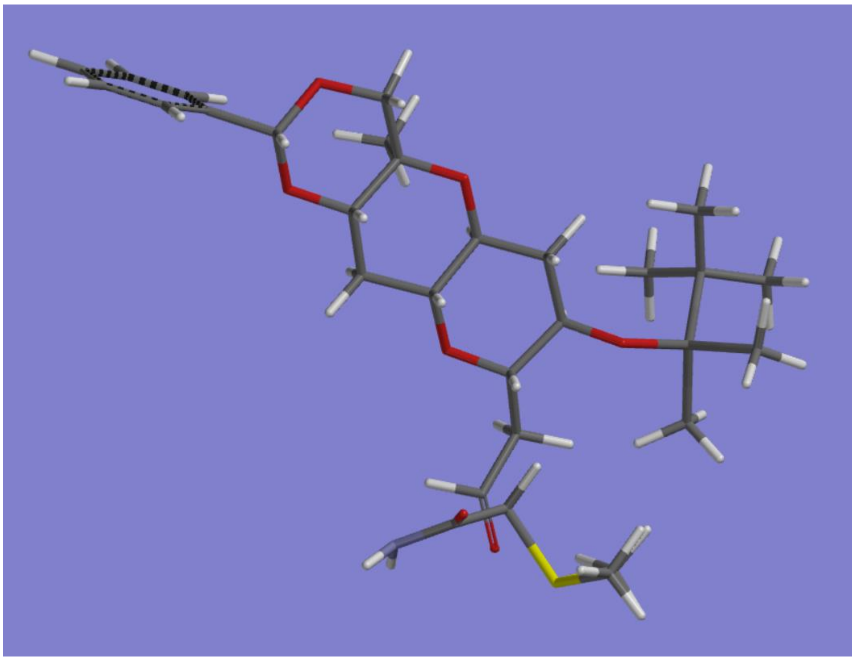

Figure S12. Transition state **[B]**$^{\neq}$ (Approach Ylide *si*-Aldehyde *si*)

Atom coordinates (TS *si-si* **[B]**$^{\neq}$):

H	-4.170138	3.402398	1.382156
C	-4.161762	2.304272	1.478575
C	-5.770250	0.562053	1.137581
C	-3.526344	0.094969	0.595567
C	-3.177324	1.614017	0.501412
H	-5.651245	0.143592	2.170431
H	-3.942432	2.046340	2.532756
C	-3.344978	2.205580	-0.902470
H	-4.316332	1.930596	-1.335053
H	-2.556123	1.853572	-1.587661
H	-3.287108	3.301772	-0.876547
O	-1.841244	1.810011	0.990149
H	-3.395613	-0.240901	1.658915
C	-2.596859	-0.738866	-0.283933
H	-2.835351	-1.816809	-0.209057
H	-2.705339	-0.480673	-1.356126
C	-0.894656	1.076834	0.205920
C	-1.177978	-0.450462	0.228475
H	-1.067177	-0.847110	1.269483
O	-0.251153	-1.108045	-0.624939
C	0.497097	1.319164	0.777901
H	0.544883	1.014450	1.842996
H	-0.955055	1.454461	-0.851379
C	-7.176894	0.383034	0.612098
C	-9.777629	0.035505	-0.320208
C	-8.132692	-0.231980	1.421076
C	-7.525577	0.823782	-0.664537
C	-8.825570	0.648527	-1.127367
C	-9.430453	-0.404152	0.953195
H	-7.863868	-0.580400	2.424960
H	-6.771295	1.306489	-1.298209
H	-9.098033	0.994341	-2.129731
H	-10.179692	-0.887018	1.588757
H	-10.800191	-0.101575	-0.686394
O	-4.888254	-0.101801	0.231581

O	-5.494918	1.961871	1.146874
H	0.747490	2.402866	0.766124
C	1.524233	0.529623	-0.063306
C	1.101605	-0.974341	-0.178282
H	1.205315	-1.461291	0.830114
H	1.583254	0.977834	-1.092120
O	2.760200	0.589858	0.593879
Si	4.081095	1.467621	-0.046436
C	5.193919	1.687901	1.478145
H	4.803046	2.461619	2.145356
H	6.206117	1.977124	1.180771
H	5.259766	0.755693	2.045777
C	3.687352	3.201990	-0.838159
C	2.897303	2.989881	-2.108654
H	1.953523	2.453469	-1.912121
H	3.465042	2.397713	-2.840393
H	2.638665	3.944736	-2.588030
C	2.911406	4.021395	0.166129
H	1.937887	3.560080	0.402169
H	2.708591	5.034139	-0.211294
H	3.463736	4.127928	1.110497
C	5.025549	3.843444	-1.135028
H	5.613913	3.242240	-1.843004
H	5.625354	3.960398	-0.220984
H	4.903118	4.842363	-1.577909
C	1.952695	-1.726940	-1.204033
H	3.007125	-1.377254	-1.123161
H	1.641998	-1.463444	-2.235352
C	4.990877	0.374369	-1.308807
H	5.886172	-0.070125	-0.864395
H	5.301622	0.945275	-2.188830
H	4.344227	-0.446018	-1.651296
C	1.886360	-3.272984	-1.070246
H	0.836659	-3.589560	-1.280542
O	2.767311	-3.909095	-1.858326
C	2.287417	-3.707359	0.403825
H	2.342122	-2.815416	1.077049
S	3.979907	-4.482035	0.149678
C	4.643326	-4.219362	1.814240
H	5.635093	-4.679648	1.899957
H	3.995855	-4.694266	2.565261
H	4.745168	-3.165164	2.102234
C	5.022741	-3.343534	-0.790898
H	4.420039	-3.210138	-1.744095
H	5.987881	-3.807138	-1.006531
H	5.181849	-2.362931	-0.330356
C	1.396929	-4.749827	1.059904
N	0.597685	-5.594193	0.282261
H	0.235116	-6.405084	0.726960
H	0.862954	-5.711911	-0.668572
O	1.301173	-4.848338	2.283582

This transition state, also confirmed by the presence of a single imaginary vibration mode at the frequency of –254 cm^{-1}, presented an energy of –1141.518 KJ/mol, and was similarly reached from the minimun energy conformer.

According to these theoretical calculations, transition state **[A]**$^{\neq}$ resulted with less energy, compared with transition state **[B]**$^{\neq}$, which resulted consistent with the experimental result. Nevertheless, the energy difference between both transition states was only of 3.42 Kcal/mol. This energy difference would determine a ratio of the corresponding rate constants of 0.993 at 0ºC, indicating that the reactions involving each transition state would proceed at similar rates. A more detailed and complex theoretical calculation, including a higher level of theory and solvatation models could provide major differences between the energies of the transition states **[A]**$^{\neq}$ and **[B]**$^{\neq}$ that justify the observed experimental result. Anyway, this first approach, by using a semiempirial method (PM3) in vacuo, has provided an energetic difference in favour of the transition state **[A]**$^{\neq}$.

3. ^{1}H and ^{13}C NMR Spectra of Compounds

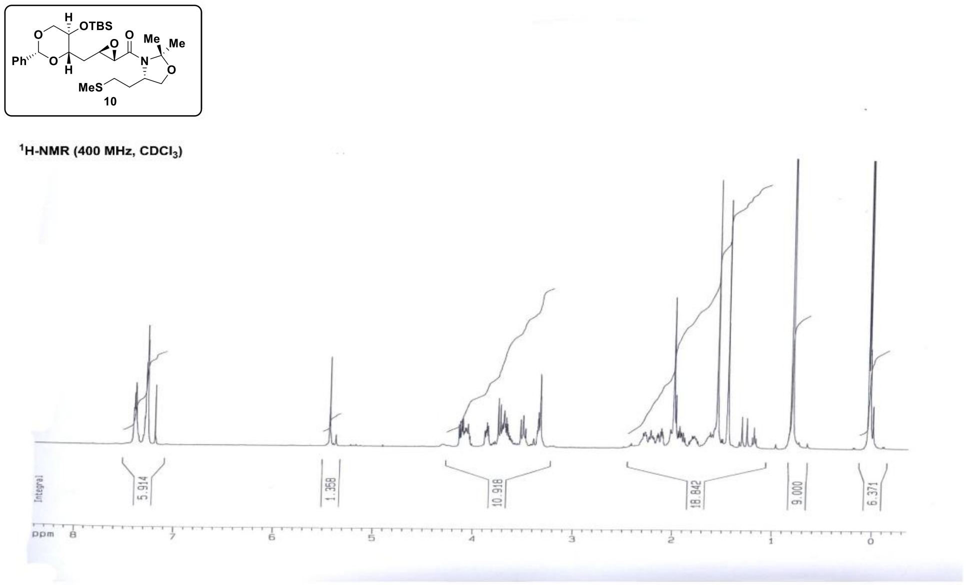

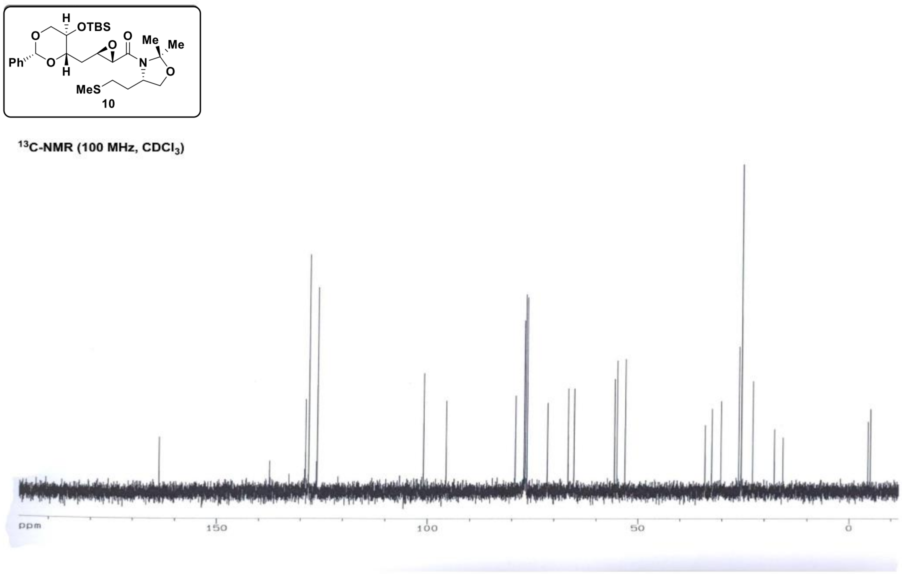

Figure S13. ^{1}H and ^{13}C-NMR spectra of compound **10**

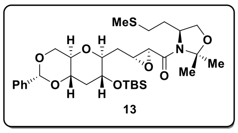

^{1}H-NMR (400 MHz, $CDCl_3$)

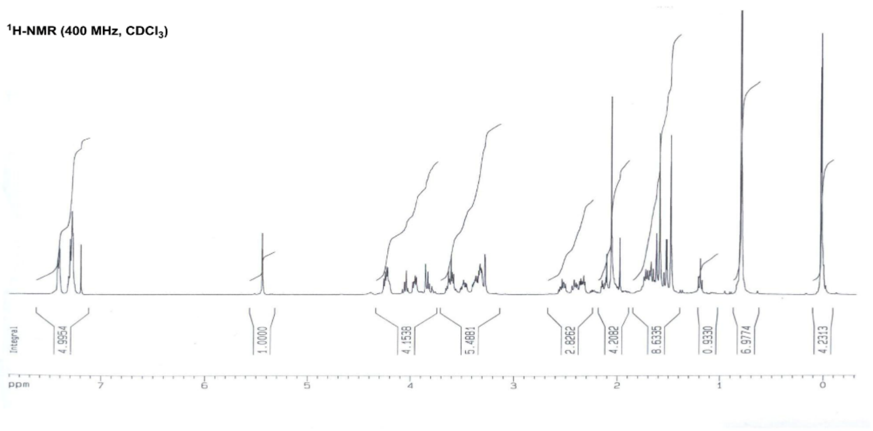

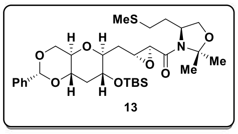

^{13}C-NMR (100 MHz, $CDCl_3$)

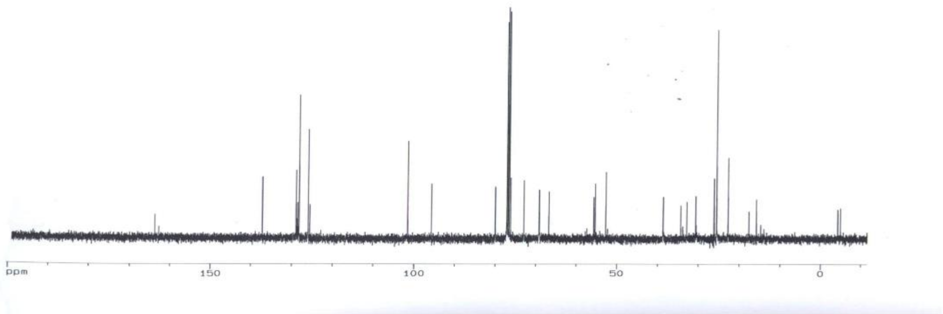

Figure S14. ^{1}H and ^{13}C-NMR spectra of compound **13**

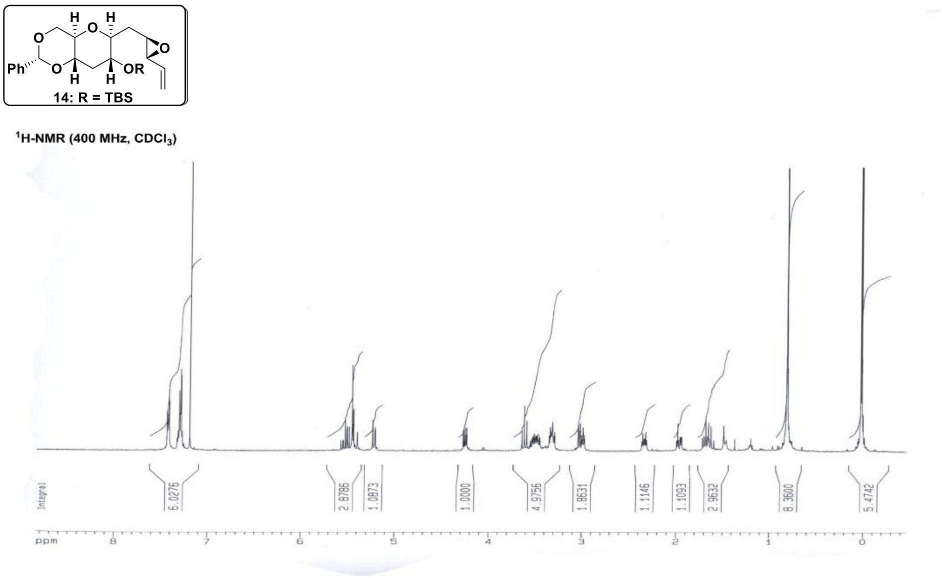

Figure S15. ^{1}H-NMR spectra of compound **14**

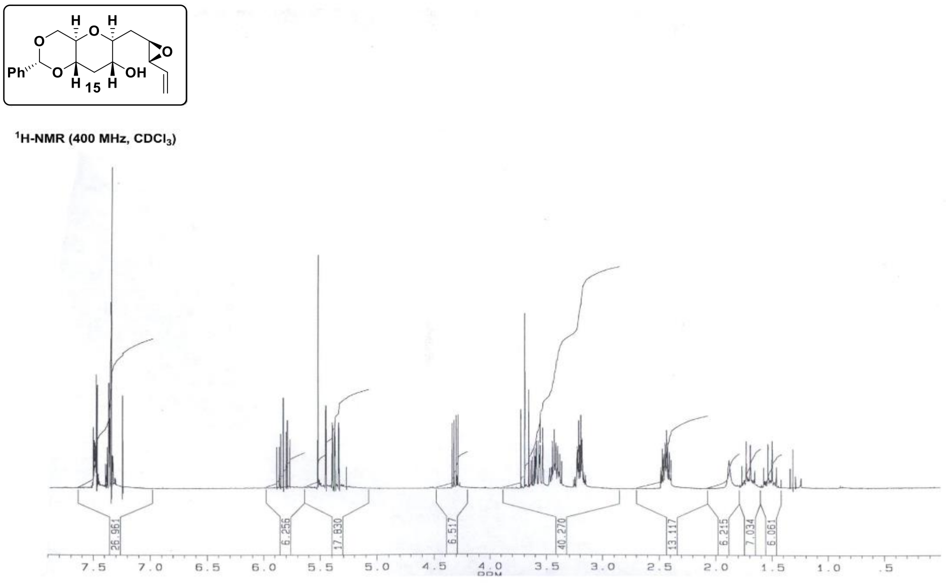

Figure S16. ^{1}H-NMR spectra of compound **15**

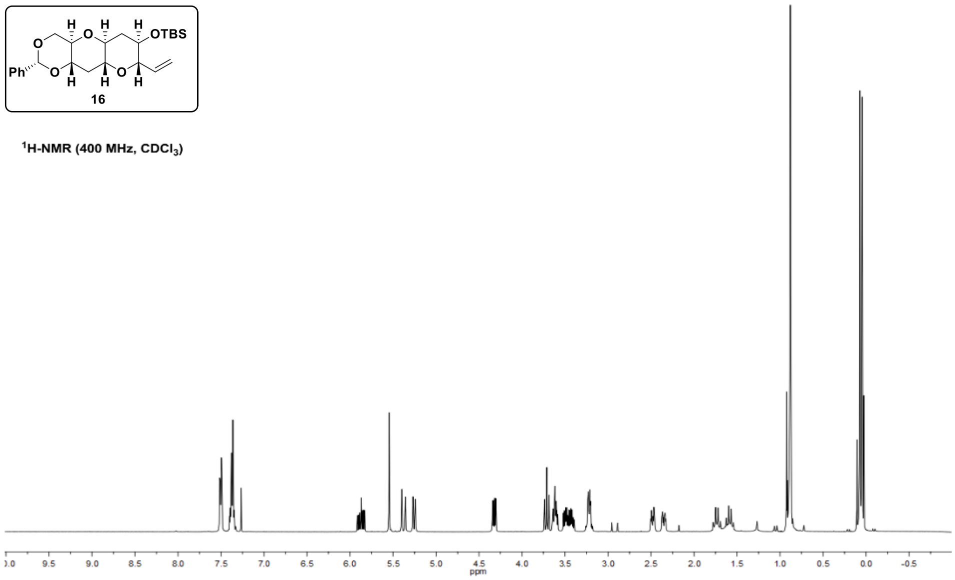

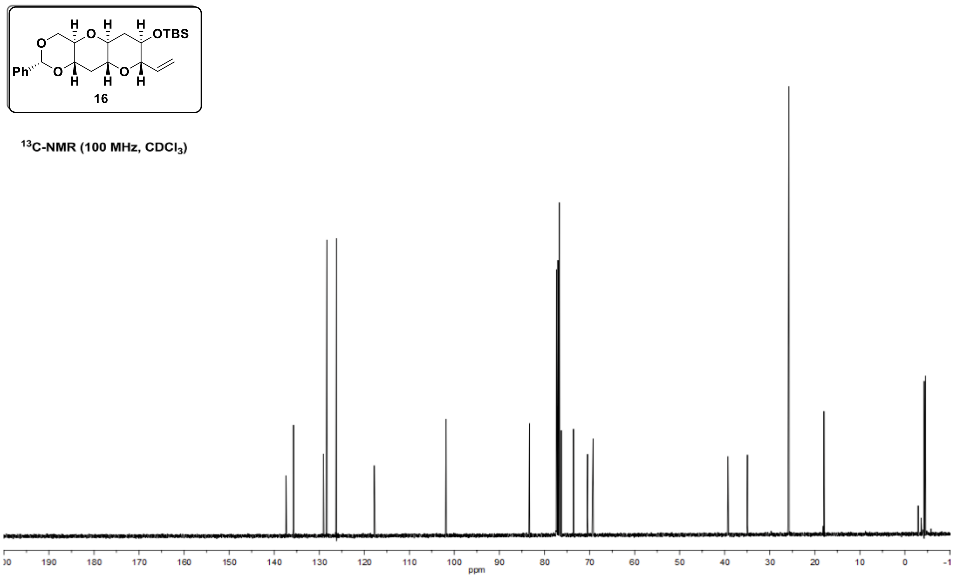

Figure S17. ^{1}H and ^{13}C-NMR spectra of compound **16**

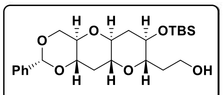

17

^{1}H-NMR (400 MHz, $CDCl_3$)

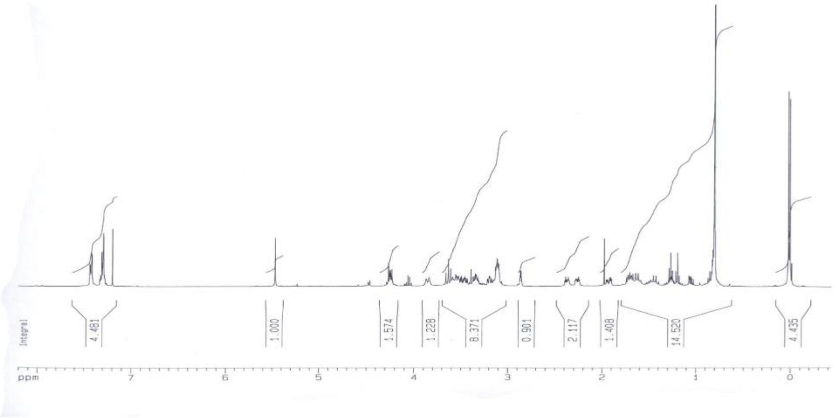

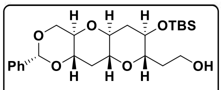

17

^{13}C-NMR (100 MHz, $CDCl_3$)

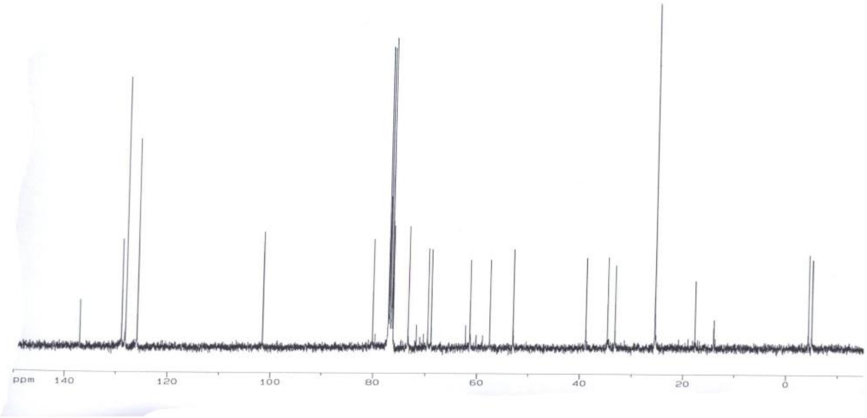

Figure S18. ^{1}H and ^{13}C-NMR spectra of compound **17**

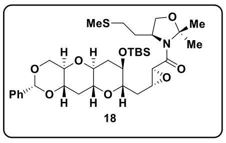

^{1}H-NMR (400 MHz, $CDCl_3$)

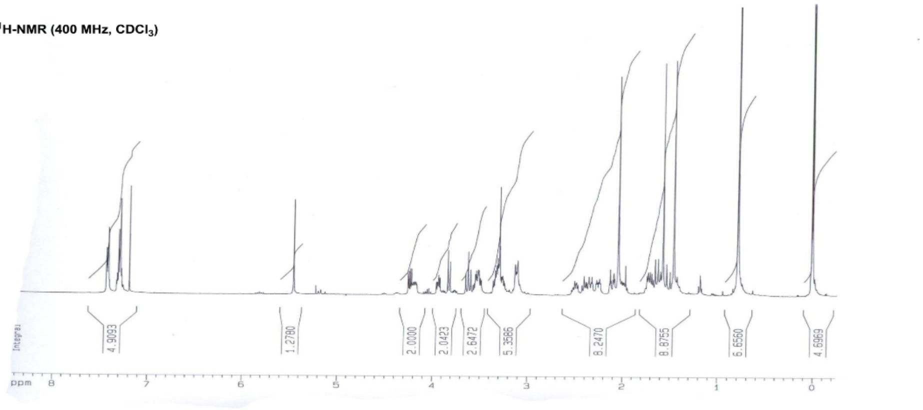

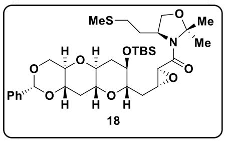

^{13}C-NMR (100 MHz, $CDCl_3$)

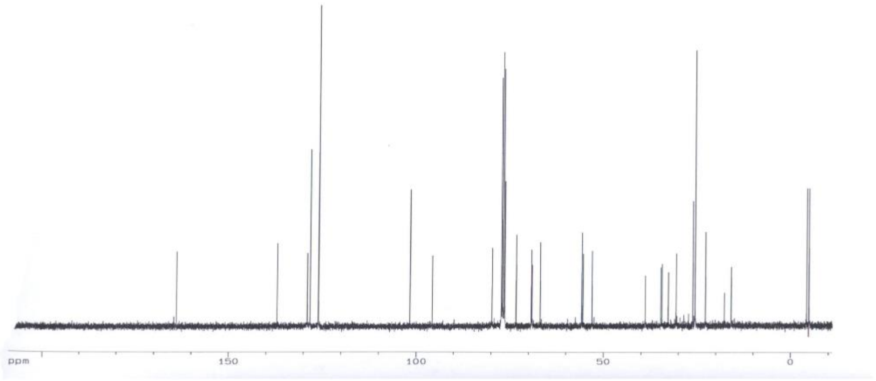

Figure S19. ^{1}H and ^{13}C-NMR spectra of compound **18**

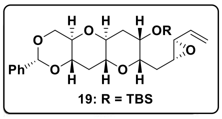

^{1}H-NMR (400 MHz, $CDCl_3$)

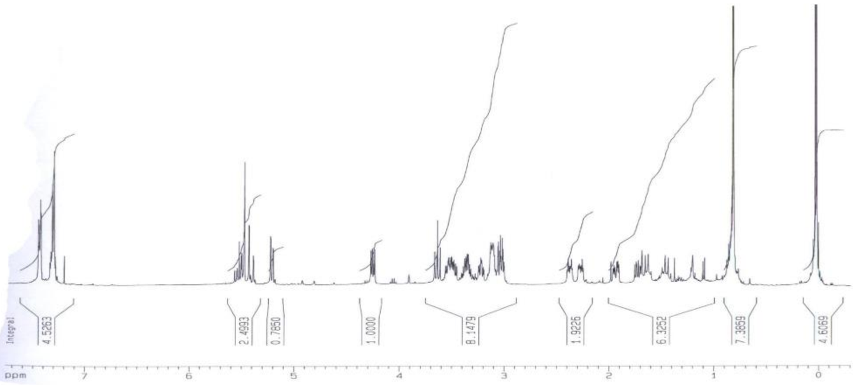

Figure S20. ^{1}H-NMR spectra of compound **19**

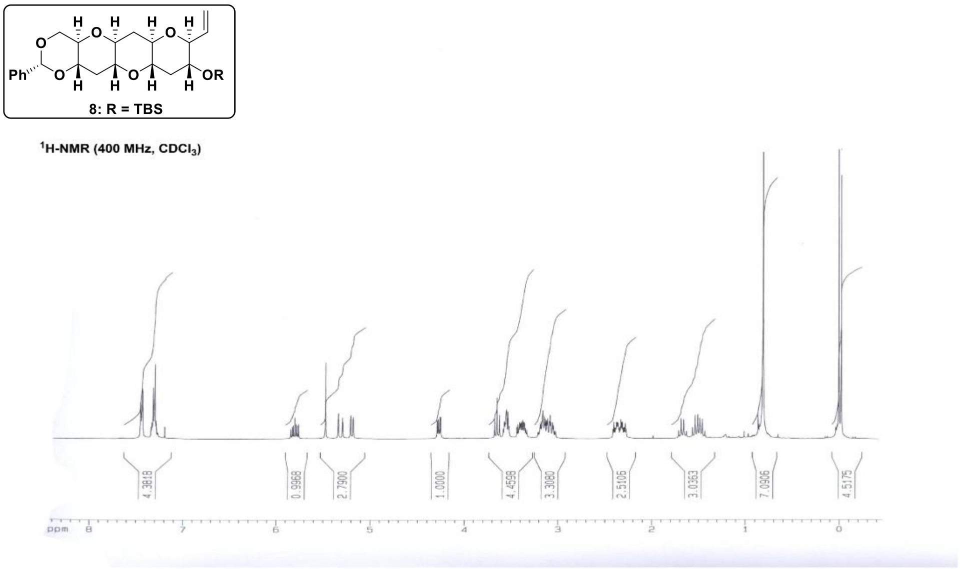

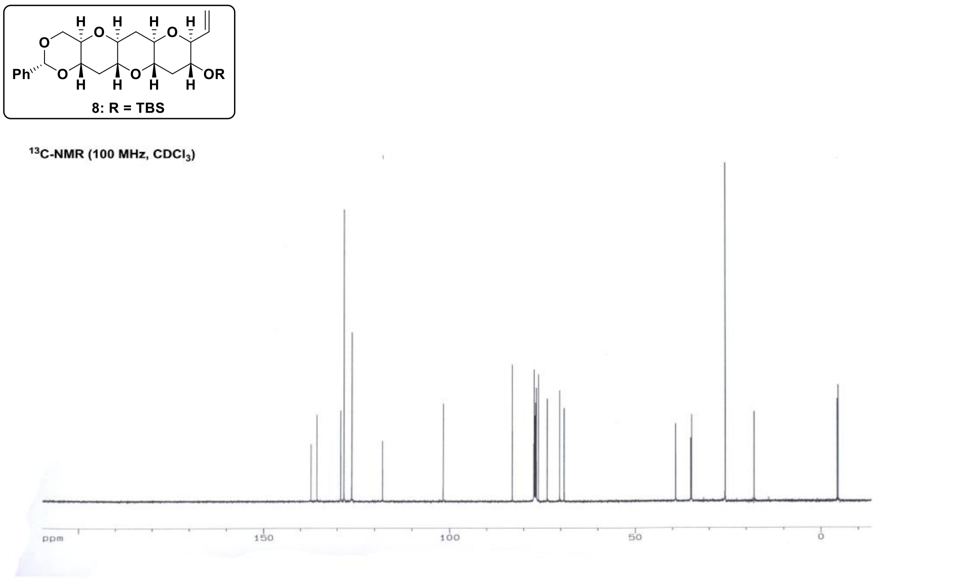

Figure S21. ^{1}H and ^{13}C-NMR spectra of compound **8**

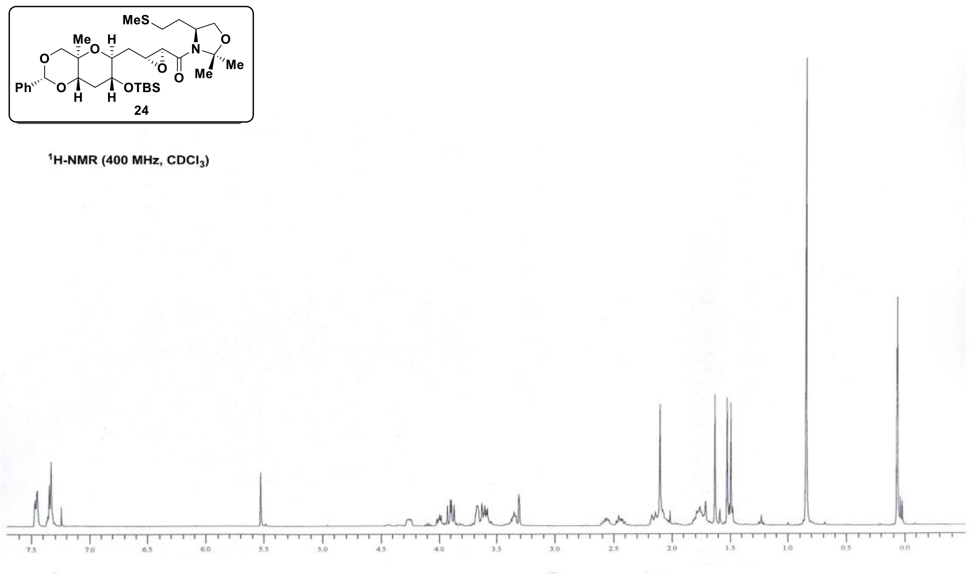

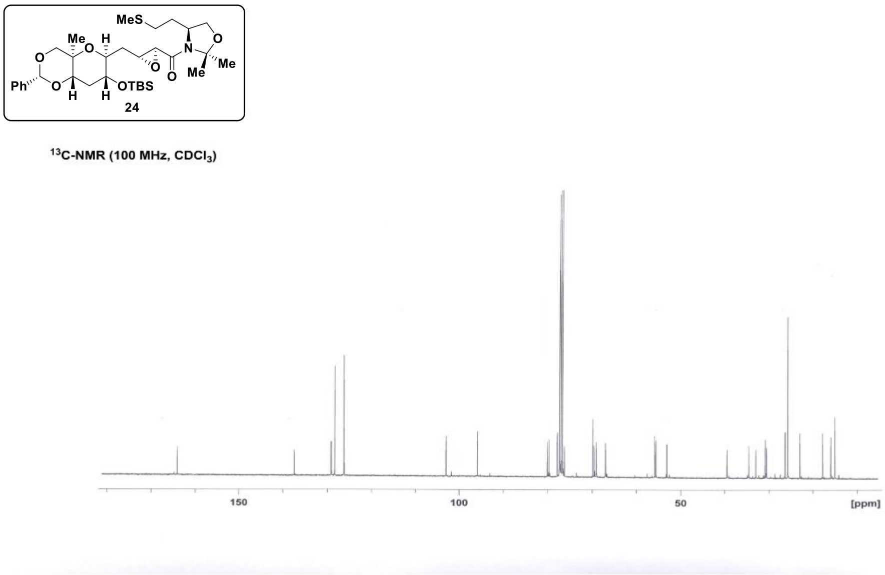

Figure S22. ^{1}H and ^{13}C-NMR spectra of compound **24**

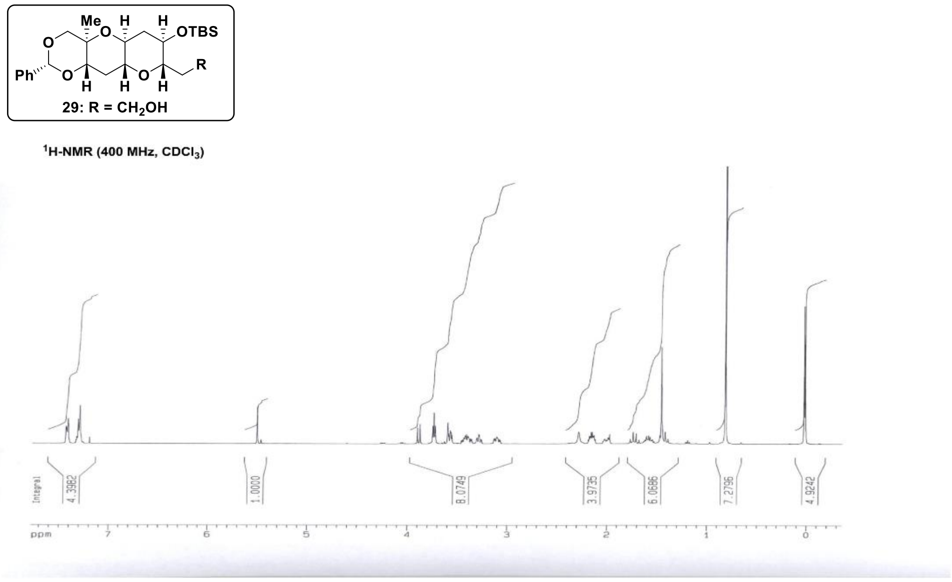

Figure S23. ^{1}H-NMR spectra of compound **29**

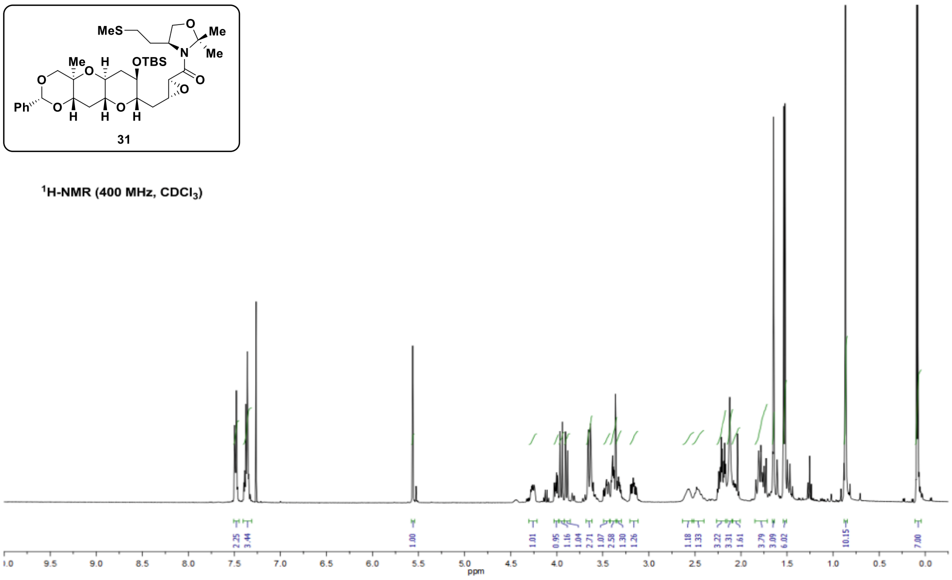

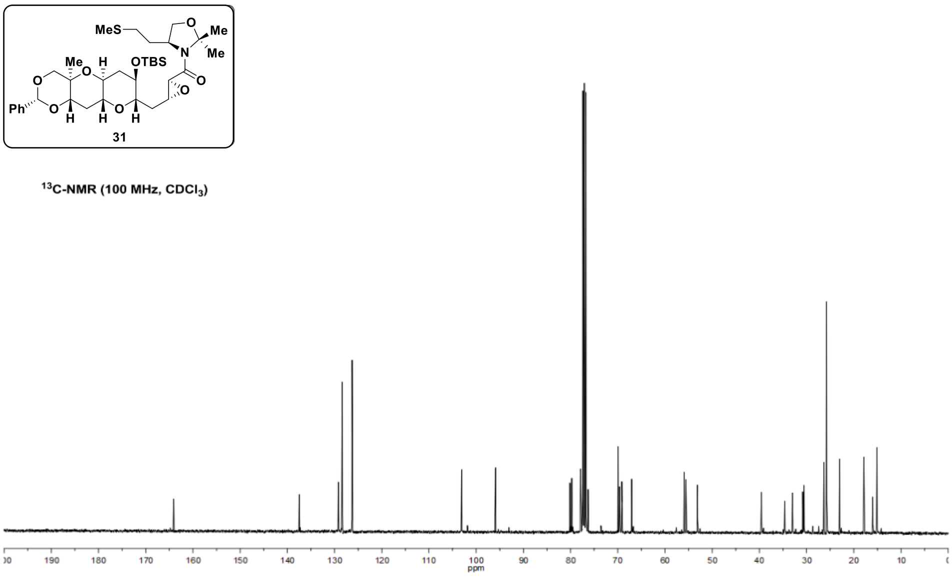

Figure S24. ^{1}H and ^{13}C-NMR spectra of compound **31**

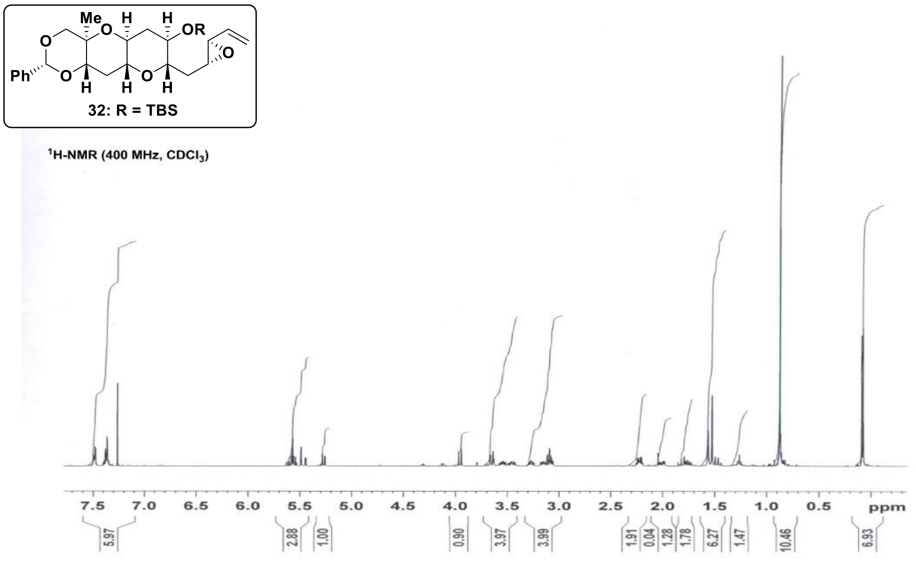

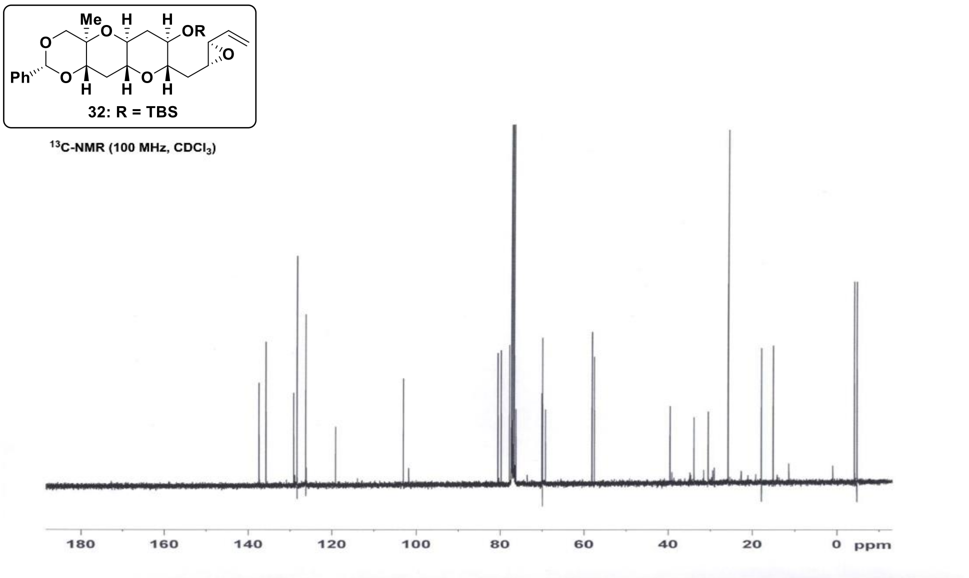

Figure S25. ^{1}H and ^{13}C-NMR spectra of compound **32**

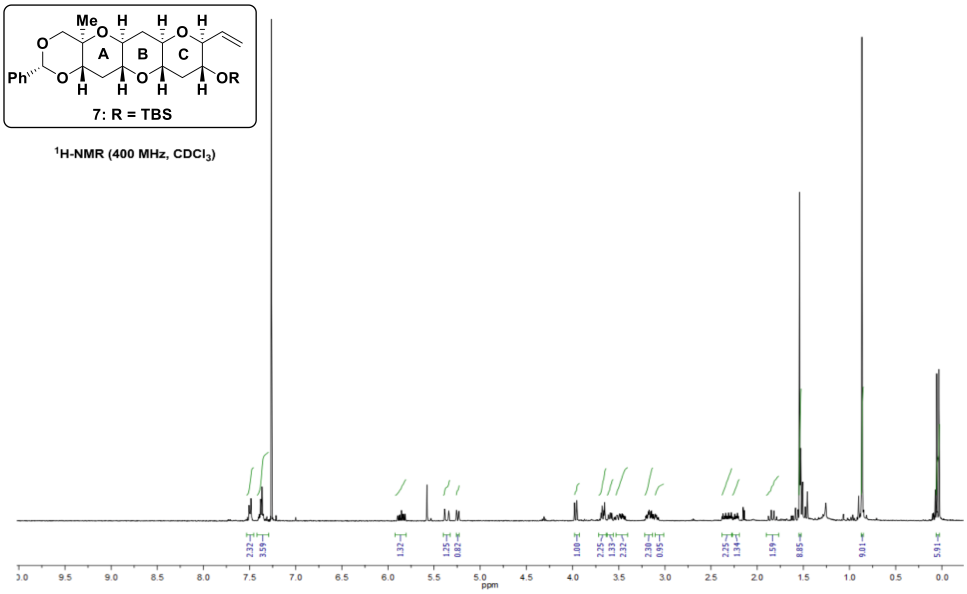

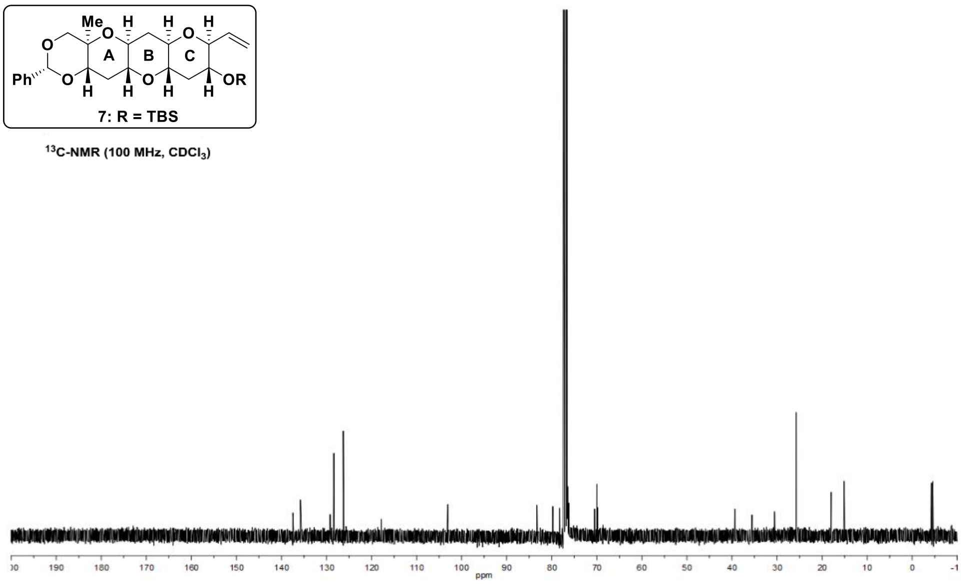

Figure S26. ^{1}H and ^{13}C-NMR spectra of compound **7**
